# *Salvia officinalis* L. exerts oncostatic effects in rodent and *in vitro* models of breast carcinoma

**DOI:** 10.3389/fphar.2024.1216199

**Published:** 2024-02-23

**Authors:** Peter Kubatka, Alena Mazurakova, Lenka Koklesova, Tomas Kuruc, Marek Samec, Karol Kajo, Klaudia Kotorova, Marian Adamkov, Karel Smejkal, Emil Svajdlenka, Dana Dvorska, Dusan Brany, Eva Baranovicova, Vladimira Sadlonova, Jan Mojzis, Martin Kello

**Affiliations:** ^1^ Department of Histology and Embryology, Jessenius Faculty of Medicine, Comenius University in Bratislava, Martin, Slovakia; ^2^ Department of Anatomy, Jessenius Faculty of Medicine, Comenius University in Bratislava, Martin, Slovakia; ^3^ Department of Pharmacology, Faculty of Medicine, P. J. Šafárik University, Košice, Slovakia; ^4^ Department of Medical Biology, Jessenius Faculty of Medicine, Comenius University in Bratislava, Martin, Slovakia; ^5^ Department of Pathology, St. Elisabeth Oncology Institute, Bratislava, Slovakia; ^6^ Department of Natural Drugs, Faculty of Pharmacy, Masaryk University, Brno, Czechia; ^7^ Biomedical Centre Martin, Jessenius Faculty of Medicine, Comenius University in Bratislava, Martin, Slovakia; ^8^ Department of Microbiology and Immunology, Jessenius Faculty of Medicine, Comenius University in Bratislava, Martin, Slovakia

**Keywords:** *Salvia officinalis* L., apoptosis, breast carcinoma, cancer stem cells, cell proliferation, epigenetics, human carcinoma cell lines, inflammatory cytokines

## Abstract

**Introduction:** Based on extensive data from oncology research, the use of phytochemicals or plant-based nutraceuticals is considered an innovative tool for cancer management. This research aimed to analyze the oncostatic properties of *Salvia officinalis* L. *[Lamiaceae; Salviae officinalis herba]* using animal and *in vitro* models of breast carcinoma (BC).

**Methods:** The effects of dietary administered *S. officinalis* in two concentrations (0.1%/SAL 0.1/and 1%/SAL 1/) were assessed in both syngeneic 4T1 mouse and chemically induced rat models of BC. The histopathological and molecular evaluations of rodent carcinoma specimens were performed after the autopsy. Besides, numerous *in vitro* analyses using two human cancer cell lines were performed.

**Results and Conclusion:** The dominant metabolites found in *S. officinalis* propylene glycol extract (SPGE) were representatives of phenolics, specifically rosmarinic, protocatechuic, and salicylic acids. Furthermore, the occurrence of triterpenoids ursolic and oleanolic acid was proved in SPGE. In a mouse model, a non-significant tumor volume decrease after S. *officinalis* treatment was associated with a significant reduction in the mitotic activity index of 4T1 tumors by 37.5% (SAL 0.1) and 31.5% (SAL 1) vs. controls (set as a blank group with not applied salvia in the diet). In addition, salvia at higher doses significantly decreased necrosis/whole tumor area ratio by 46% when compared to control tumor samples. In a rat chemoprevention study, *S. officinalis* at a higher dose significantly lengthened the latency of tumors by 8.5 days and significantly improved the high/low-grade carcinomas ratio vs. controls in both doses. Analyses of the mechanisms of anticancer activities of *S*. *officinalis* included well-validated prognostic, predictive, and diagnostic biomarkers that are applied in both oncology practice and preclinical investigation. Our assessment *in vivo* revealed numerous significant changes after a comparison of treated vs. untreated cancer cells. In this regard, we found an overexpression in caspase-3, an increased Bax/Bcl-2 ratio, and a decrease in MDA, ALDH1, and EpCam expression. In addition, salvia reduced TGF-β serum levels in rats (decrease in IL-6 and TNF-α levels were with borderline significance). Evaluation of epigenetic modifications in rat cancer specimens *in vivo* revealed a decline in the lysine methylations of *H3K4m3* and an increase in lysine acetylation in *H4K16ac* levels in treated groups. Salvia decreased the relative levels of oncogenic miR21 and tumor-suppressive miR145 (miR210, miR22, miR34a, and miR155 were not significantly altered). The methylation of *ATM* and *PTEN* promoters was decreased after *S. officinalis* treatment (*PITX2, RASSF1*, and *TIMP3* promoters were not altered). Analyzing plasma metabolomics profile in tumor-bearing rats, we found reduced levels of ketoacids derived from BCAAs after salvia treatment. *In vitro* analyses revealed significant anti-cancer effects of SPGE extract in MCF‐7 and MDA-MB-231 cell lines (cytotoxicity, caspase‐3/-7, Bcl‐2, Annexin V/PI, cell cycle, BrdU, and mitochondrial membrane potential). Our study demonstrates the significant chemopreventive and treatment effects of salvia haulm using animal or *in vitro* BC models.

## Graphical Abstract

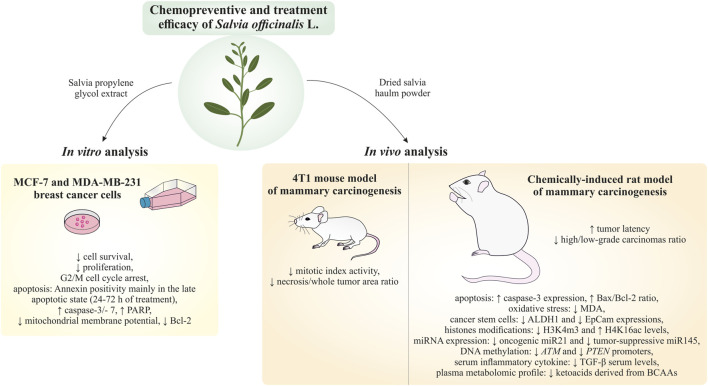

## 1 Introduction

Epidemiological studies as well as the data from preclinical research have documented that phytochemicals from dietary sources like fruits, vegetables, medicinal plants, and tea exert beneficial effects on organisms and their systematic and long-term intake (4–5 times a week for several years) is linked with reduced cancer risk (Kubatka et al., 2015; Kubatka et al., 2016a; Pourzand et al., 2016; Mao et al., 2017). Notably, the protective effects of phytochemicals are substantially associated with reducing oxidative stress and inflammation (Kubatka et al., 2021). Anticancer characteristics of phytochemicals and their derivatives are exerted by their acting as potent cell signaling modulators. Phytochemicals have exhibited significant oncostatic activities by restraining the initiation and progression of malignancy by affecting mechanisms such as cell cycle, programmed cell death, neovascularization, the activity of stem cells, and metastasis (Abotaleb et al., 2018; Liskova et al., 2019; Liskova et al., 2020a; Liskova et al., 2020b; Tesfaye et al., 2020; Brockmueller et al., 2021). Importantly, phytochemicals demonstrate potent anticancer activities *via* epigenetic modulation, which include chemical modifications of histones, and expression changes in microRNA, oncogenes, and tumor-suppressor genes (Zwergel et al., 2016; Samec et al., 2019a; Samec et al., 2019b; Jasek et al., 2019).

Medicinal plants have great potential due to their pleiotropic effects on the organism. *Salvia officinalis* L. [*Lamiaceae; Salviae officinalis herba]*is the typical medicinal plant in the Mediterranean and Middle East regions, which has been recently naturalized in almost the whole world. *S*. *officinalis* L. is rich in phytochemical cocktail containing essential oils based on monoterpene derivatives (camphor, cineol, borneol derivatives, thujone) and sesquiterpenes (caryophyllene); the plant commonly contains labiate tannins (rosmarinic acid, salvianolic acid), diterpenoid bitter substances carnosol derivatives), and flavonoids (cirsiliol, luteolin and apigenin derivatives, and phenolic glycosides). In traditional medicine, *S. officinalis* has been applied in the management of various types of pathologies, such as inflammation, rheumatism, gout, ulcers, diarrhea, tremor, paralysis, seizure, hyperglycemia, and dizziness (Ghorbani and Esmaeilizadeh, 2017). Anti-oxidant and anti-inflammatory characteristics of *S*. *officinalis* (Bonesi et al., 2017) also correlate with its anti-cancer properties that were documented by numerous studies. *S. officinalis* ethanol and acetone extracts demonstrated antiproliferative effects on hepatocellular carcinoma cells by evaluating cell viability, apoptosis, cell morphology, cellular ATP, and lactate dehydrogenase levels (Jiang et al., 2017). In this regard, the half inhibition concentration (IC50) values (μg/mL) of extracts from *S. officinalis* leaves on HepG2 and WRL-68 cells after 48 h of treatment were 64.4 and 67.1 in methanol extract and 90.0 and 87.5 in acetone extract. Moreover, *S. officinalis* suppressed the cell cycle and induced programmed cell death of human colorectal cell lines through the downregulation of the MAPK/ERK pathway (Jiang et al., 2017). Another study found that *S. officinalis* methanolic extract blocks the proliferation of lymphoma and leukemic cell lines through induced cell death (Zare Shahneh et al., 2013). More specifically, extract at 50–800 μg/mL in a dose and time-dependent manner decreased the proliferation of KG-1A, U937, and Raji cells by more than 80% (*p* < 0.01). IC_50_ values showed ascending order after 24 h treatment: KG-1A (214.377 μg/mL), U937 (229.312 μg/mL), and Raji (239.692 μg/mL) when compared to paclitaxel. Moreover, Yanagimichi et al. (Yanagimichi et al., 2021) reported that cirsiliol, luteolin, and carnosol represent significant inhibitory compounds of *S. officinalis* in STAT3 cell signaling in HepG2 cells. Carnosol, at 30 μM, reduced phosphorylated STAT3 by about 80% but at a dose of 90 μM downregulated also the total STAT3 level. HepG2 cells treated with luteolin at 30 μM did not show changes in phosphorylated/total STAT3 but a higher dose of 90 μM suppressed phosphorylated STAT3 in comparison with total STAT3. Cells treated with cirsiliol at 90 μM did not show a decrease in phosphorylated and total STAT3, however, authors described clear downregulation of STAT3-responsive reporter expression. In addition, *S. officinalis* ethyl acetate extract and its constituents (carnosic acid and carnosol) inhibited PGE_2_ accumulation by directly targeting mPGES-1 in a cell-free assay and thus may crucially contribute to its anti-cancer characteristics (Bauer et al., 2012). Component evaluations found that the diterpenes carnosic acid and carnosol as potential bioactive molecules decreased mPGES-1 activity with IC_50_ values of 5.0 μM.

The hypothesis of this study was based on the knowledge that the application of appropriate plant nutraceuticals rich in specific phytochemicals with the potential additive or synergistic mode of action can show significant antitumor effects, as our group has already demonstrated in the past (Kubatka et al., 2015; Kubatka et al., 2016a; Kubatka et al., 2016b; Kubatka et al., 2017a; Kubatka et al., 2017b; Kubatka et al., 2019; Kubatka et al., 2020a; Kubatka et al., 2020b). This study aimed to analyze the anticancer activities of *S. officinalis* haulm in therapeutic (allograft) and chemopreventive breast carcinoma (BC) animal models. In the mechanistic analyses, we evaluated clinically validated cancer biomarkers of cell death, proliferation, vascularization, inflammation/oxidative stress, stem cells, and epigenetic and plasma metabolomic changes. Moreover, histopathological features of tumor specimens such as the high/low-grade carcinomas ratio, tumor necrosis ratio, and mitotic activity index were assessed. Finally, to deepen the mode of anticancer action of *S. officinalis*, the parameters of cell growth and cell deaths were performed using human hormone-sensitive MCF-7 and metastatic triple-negative MDA-MB-231 BC cell lines.

## 2 Material and methods

### 2.1 Rodent models

Female BALB/c mice (Velaz, Prague, Czech Republic) aged 10 weeks and weighing between 17–19 g and female Sprague-Dawley rats (Charles River Laboratories, Sulzfeld, Germany) aged 5 weeks and weighing between 125–140 g were used in the study. Rats were acclimated to a controlled vivarium environment that included a 12-h artificial light cycle, a 23°C ± 2°C temperature range, and a 40%–60% relative humidity range. The animals were fed *ad libitum* with a Ssniff® diet (R-Z/M-Z low-phytoestrogen; Soest, Germany) and were provided with unlimited access to water. N-nitroso-N-methylurea (NMU, Sigma, Deisenhofen, Germany) was applied to induce mammary gland cancer in rats. Specifically, the carcinogen was injected intraperitoneally (50 mg/kg as a single dosage) at 42. postnatal day. The factor significantly increasing the inducibility of mammary carcinogenesis in the Sprague-Dawley rats represents a period of carcinogen administration in early puberty during postnatal days 40–46 in comparison with the period after postnatal day 50 (Russo et al., 1990; Kubatka et al., 2002). This research method imitates premenopausal women who have a higher risk of BC etiology. A syngeneic mouse model represented the treatment BC model. To induce mammary carcinogenesis, 1 × 10^5^ of 4T1 cells per animal (mouse mammary adenocarcinoma) was subcutaneously injected into the abdomen mammary gland area (Kubatka et al., 2019; Kubatka et al., 2020a; Kubatka et al., 2020b). *Salvia officinalis* L. [*Lamiaceae; Salviae officinalis herba]* haulm powder (Calendula, Nová Ľubovňa, Slovakia; site of origin: North-East Slovakia) administration in the allograft mouse model (treatment study) began on the day that the 4T1 cells were inoculated and lasted for 15 days. In rats, *S. officinalis* administration (chemoprevention study) started 1 week before carcinogenesis induction and lasted for 14 weeks consecutive weeks. In both animal models, *S. officinalis* was applied in (a) low concentration 1 g/kg–0.1% (w/w) (SAL 0.1) and (b) high concentration 10 g/kg–1% (w/w) (SAL 1). The lower dose of salvia (0.1% in the diet) was derived from human dosing typical for the Mediterranean diet (about 2 g per day). Due to the different pharmacokinetics and pharmacodynamics of *S. officinalis* in humans and rodents, we used also 10 times higher doses in the diet in both rodent studies (1%). Moreover, the higher dose of salvia was an insurance dose, since the dosing of *S. officinalis* through diet in rodent BC models has not been published so far, and the potential effect of salvia in rodents could have been masked in the low-dose group. The haulm was processed into the diet by “cold pelleting procedure.” Mice (*n* = 78) and rats (*n* = 75) were randomly divided into three experimental groups. (1) control group without *S. officinalis* in the diet (this group was set as blank control in which the background and the diet was applied with no tested sample); (2) administration of *S. officinalis* in the diet at a lower dose (SAL 0.1); and (3) administration of *S. officinalis* in the diet at a higher dose (SAL 1). Tumor incidence represents the percentage of tumor-bearing animals per group, tumor frequency was expressed as the number of tumors evaluated for all animals in the group, and the latency period was determined by the period from the administration of the carcinogen to the appearance of the first tumor in the animal.

From the fourth day following the inoculation of 4T1 cells in mice, the tumor growth (volume) was observed three times each week. The rats were palpated each week beginning the fifth week after carcinogen application to check for each mammary tumor’s presence, size, and location (palpable if tumor diameter is greater than 0.4–0.5 cm). Within 24-hour intervals, dietary intake was monitored twice in mice and four times in rats. As a result, the average daily doses of *S. officinalis* administered to mice and rats in each group were calculated. At the end of both experiments, rodents were euthanized by quick decapitation and mammary lesions were excised and measured (Kubatka et al., 2019; Kubatka et al., 2020a; Kubatka et al., 2020b).

### 2.2 Histopathology and immunohistochemistry of rodent tumor samples

Each tissue sample of rodent (mouse and rat) carcinoma was routinely formalin-fixed and paraffin-embedded. In the mice model, the average tumor area (the count of all tumor areas divided by the number of tumors) and the average area of necrosis (the count of all necrosis divided by the number of tumors) of each tumor were determined. Subsequently, the necrosis ratio was defined as the ratio of the average area of necrosis to the average area of tumors. The area of the tumor and the extent of necrosis were established in the histological slide. If the tumor had a circular outline, the area was determined as πr^2^ (r = radius); if the tumor showed the characteristic of an ellipse, the area was determined as π x a x b (a, b = semi-axes); if the tumor showed the characteristic of a rectangle, the area was determined as a x b (a, b sides of the rectangle) and if the tumor showed the shape of a trapezoid, the area was determined as (a + c) x v/2. If the tumor reached the size of one of the high power fields (HPFs) (e.g., with ×4 objective), the area was determined based on this microscopic field. The contours of the necrosis were outlined in the histological slide and the area of the necrosis was usually determined as their extent according to the size of the HPF, or multiple of the area of the HPF (with objective 4x = 23.76 mm^2^, with objective 10x = 3.8 mm^2^), with objective 20x = 0.95 mm^2^). The smallest evaluable necrosis was its extent in one HPF (0.24 mm^2^; i.e., at magnification ×400 with a diameter of the field of view 0 .5 mm). If the necrosis was smaller than the HPF, it was evaluated as punctiform.

Tumor samples (from rats) were evaluated complexly during the microscopic examination, at first in low magnification (×40), the overall structure of the tumor was recorded, focusing on growth microarchitectural characteristics (e.g., assessment of the proportion of glandular and solid components, and necrosis for histological grading). Subsequently, detailed cellular characteristics (i.e., degree of cellular atypia and number of mitotic figures) were assessed at higher magnifications (×100 and ×200). The mitotic score (both in rats and mice) was determined by the number of mitotic figures found in 10 consecutive HPFs in the most mitotically active part of the tumor. Only identifiable mitotic figures were counted; hyperchromatic, karyorrhetic, or apoptotic nuclei were excluded. In rare cases, where the total tumor area from mice was less than the sum of 10 HPFs, the number of mitoses in five HPFs was evaluated and multiplied by two.

The criteria for standardized classification of mammary tumors were assigned for the classification of each rat mammary tumor. Rat mammary tumors were also sub-divided as low-grade or high-grade (tumor grading as an additional parameter). Also, the standard diagnostic classification method was utilized to select categorization criteria (cell atypia, solidization, index of mitotic activity, and necrosis): firstly, solidization if >30% of tumor sample shows solid growth; secondly, a high index of mitotic activity if ≥10 mitoses is observed in 10 HPFs; thirdly, necrosis if the occurrence of comedo (not infarct) was confirmed (Kubatka et al., 2012). Accordingly, high-grade carcinomas were considered tumors with ≥2 positive criteria, and low-grade carcinomas were considered tumors with ≤1 positive criterion. In mice tumor samples, the mitotic activity index and all tumor area/necrosis ratios were assessed (Kubatka et al., 2019; Kubatka et al., 2020a; Kubatka et al., 2020b).

The sample selection (the most relevant section of mammary tumor in a paraffin block, including typing characteristics) for immunohistochemical analysis was based on specific criteria: the representation (substantial) of vital tumor epithelial component without any regressive changes (e.g., necrosis). The detection of markers (precisely selected for mechanistic study) was performed *via* an indirect immunohistochemical method on whole paraffin sections with commercially available rat-specific antibodies (Thermo Fisher Scientific, Rockford, IL, USA; Santa Cruz Biotechnology, Paso Robles, CA, USA; GeneTex, Irvine, CA, USA; Dako, Glostrup, Denmark; Boster Biological Technology, Pleasanton, CA, USA; Bioss, Woburn, MA, USA; Abcam, Cambridge, MA, USA). Immunohistochemical staining was performed according to the manufacturer’s recommendations (Autostainer Link 48/Hermes/). Each primary antibody concentration was as follows: Bax 1:200 (sc-526); Bcl-2 1:200 (sc-492); cleaved caspase-3 1:500 (catalogue no. ab2302); Ki-67 1:50 (M7248 01); VEGFA 1:150 (sc-57496); VEGFR-2 1:80 (sc-6251); MDA 1:1000 (ab6463); EpCam 1:160 (ab71916); ALDH1A1 1:500 (pa532127); CD133 1:150 (ab19898); CD44 1:200 (pa1021-2); CD24 1:200 (gtx37755); H3K4m3 1:500 (ab8580); H3K9m3 1:400 (ab8898); H4K16ac 1:200 (ab109463), H4K20m3 1:300 (ab9053). The visualization of primary antibodies (with diaminobenzidine tetrahydrochloride as a substrate) was performed using the secondary staining system EnVision, Dual Link System-HRP, (Dako North America, Carpinteria, CA, USA, cat. No. K060911). The samples with the omission of primary antibodies were assigned as negative controls. The evaluation of immunohistochemically detected expression of selected antigens was performed using a precise morphometric method. Specifically, the sections were first screened and microscopically analyzed (Olympus BX41N, magnifications of digital images ×400). The quantification of protein expression was obtained as the average percentage of antigen-positive area in standard fields (0.5655 mm^2^) of tumor cells’ hot spot areas. Using the morphometric method, three hot spots were analyzed per tumor sample. The morphometric analysis of digital images was performed using QuickPHOTO MICRO software, version 3.0 (Promicra, Prague, Czech Republic). The obtained values were further compared between tumor tissue specimens from experimental groups with salvia in diet (SAL 0.1 and SAL 1) and non-treated (control) tumor tissue specimens. At least 60 tumor samples were analyzed for each marker (960 tumor slides for 16 markers) (Kubatka et al., 2019; Kubatka et al., 2020a; Kubatka et al., 2020b).

### 2.3 Analysis of miRNA expression

Total RNA was isolated from tumor tissues using a commercial miRVana microRNA isolation kit (Thermo Fisher Scientific, Waltham, MA, USA), which is in detail described in the supplementary protocol. Subsequently, the NanoDrop ND-2000 spectrophotometer (Thermo Scientific, Wilmington, Delaware, USA) was utilized for the RNA quantification, followed by reverse transcription *via* TaqMan Advanced miRNA cDNA Synthesis Kit (Applied Biosystems, Life Technologies, Carlsbad, CA, USA). The cDNA samples were kept at −20°C until further analyses. Quantitative real-time PCR was carried out using miRNA-specific TaqManTM advanced miRNA assays kit (Applied Biosystems, Life Technologies, Carlsbad, CA, USA) for the tumor-suppressive miRNA (miR-22, miR-34a, miR-145) and the oncogenic miRNA (miR-21, miR-155, and miR-210). MiR-191-5p was chosen as an internal control to normalize the quantities of cDNA in the samples by relevant publications and protocol recommendations. After that, quantitative real-time PCR was performed using the AB7500 real-time system from Applied Biosystems, Life Technologies, Carlsbad, California, USA. Each qPCR reaction was utilized in duplicates and the resultant Cq values were then averaged (Kubatka et al., 2019; Kubatka et al., 2020a; Kubatka et al., 2020b). Fold changes were calculated in Excel using the standard ∆∆Cq method.

A list of catalogue numbers and specific ID’s of used miRNA assays.

**Table:** 

Assay’s name	Catalog numbers	Specific ID
rno-miR-155-5p	A25576	002571
rno-miR-145-5p	A25576	002278
rno-miR-21-5p	A25576	000397
rno-miR-210-3p	A25576	000512
rno-miR-22-5p	A25576	002301
rno-miR-34a-5p	A25576	000426
hsa-miR-191-5p	A25576	002299

### 2.4 DNA extraction and bisulfite conversion

Homogenization and disruption of fresh frozen tissue were provided using the mechanical disruptor TissueLyser LT (Qiagen, Germany) designed for low-to-medium disruption of different types of tissue. In the first step, we placed a tissue sample (∼100 mg) and a 5 mm stainless steel bead into a precooled 2 mL round bottom tube. Subsequently, animal tissue was homogenized in 200 mL lysis buffer at 50 Hz for 1 min. In the next step, we added 20 proteinase K for the digestion of the sample and incubated it at 56°C overnight. Purification of total DNA from animal tissue was mediated using a DNeasy blood and tissue kit (Qiagen, Germany) according to the manufacturer’s protocol. The concentration of the samples was determined by Qubit™ 3.0 fluorometer (Thermo Fisher Scientific) with Qubit dsDNA BR assay kit (Thermo Fisher Scientific). Subsequently, isolated DNA (at a concentration of at least 50 ng/μL) was bisulfite-treated using an EpiTect bisulfite kit (Qiagen, Germany) according to the manufacturer’s protocol (Kubatka et al., 2019; Kubatka et al., 2020a; Kubatka et al., 2020b).

### 2.5 CpG assays and determination of methylation status by pyrosequencing

For the pyrosequencing analysis of selected regions of target genes: PTEN (6 CpG sites), TIMP3 (3 CpG sites), RASSF1A (3 CpG sites), ATM (4 CpG sites), and PITX2 (5 CpG sites), we used commercially available CpG assays (PyroMark CpG Assay, Qiagen, Germany). Primer sequences are available in the supplementary material of the manuscript. Briefly, bisulfite-converted DNA was used as a template and amplified using a PyroMark PCR kit (Qiagen, Germany). PCR conditions were as follows: initial denaturation of DNA for 15 min at 95°C, 45 cycles (96°C for 30 s, 56°C for 30 s, and 72°C for 30 s) followed by final extension at 72 °C for 10 min. PCR products were visualized using gel electrophoresis (1.75% agarose gel). The PCR products were further processed according to the manufacturer’s protocols and analyzed by PyroMark Q96 ID System (Qiagen, Germany) and PyroMark Gold Reagents (Qiagen, Germany). PyroMark Q96 software version 2.5.8 (Qiagen, Germany) automatically calculated the methylation level of selected CpG dinucleotides (Kubatka et al., 2019; Kubatka et al., 2020a; Kubatka et al., 2020b).

A list of catalogue numbers and specific ID’s of used assays.

**Table:** 

Assay’s name	Catalog numbers	Specific ID
Rn_Pten_03	978746	PM00450450
Rn_Npat_02	978746	PM00592487
Rn_Pitx2_08	978746	PM00519141
Rn_Timp3_06	978746	PM00574896
Mm_Rassf1_G2_PM	978746	PM00416297

### 2.6 Serum cytokine levels in rats

Blood samples (after clot formation) were centrifuged at 2,000 x g for 10 min. Consequently, serum samples were collected and evaluated for the following cytokines: IL-6, IL-10, TNF-α, and TGF-β. The levels of cytokines were analyzed using ELISA *in vitro* kits (Abcam, Cambridge, MA, USA) designed for quantitative measurement of cytokines in rat serum: Rat IL-6 *in vitro* ELISA Kit (ab234570), Rat IL-10 *in vitro* ELISA Kit (ab214566), Rat TNF-α *in vitro* ELISA Kit (ab236712), and Rat TGF-β *in vitro* ELISA Kit (ab119558) (Molitorisova et al., 2021).

### 2.7 Plasma metabolome profiling in tumor-bearing rats

EDTA-coated tubes were used to collect blood samples, which were centrifuged at 4°C and 2,000 rpm (380 g-forces) for 20 min. Consequently, plasma was stored at −80°C until further analysis. Plasma denaturation (Nagana Gowda et al., 2015) and further processing were carried out as follows: data acquisition, handling, and evaluation were performed according to the study by Baranovicova et al. (Baranovicova et al., 2018). We used plasma from tumor-bearing rats, only a few samplings were omitted due to insufficient amount, hemolysis, or blood clotting. The usual labeling of BCAAs for branched-chain amino acids -leucine, isoleucine, and valine, further BCKAs for their 2-oxoderivates, also branched-chain keto acids - ketoleucine (2-oxoisocaproate), ketosoleucine (3-methyl-2-oxopentanonate), and finally ketovaline (2-oxoisovalerate) was applied (Baranovicova et al., 2018).

### 2.8 Cell lines, cell cultures, and experimental design

BC cell lines MCF-7 (ER^+^, PR^+^, HER2^−^) and MDA-MB-231 (ER^−^, PR^−^, HER2^−^) (both lines: American Type Culture Collection, ATCC; Manassas, VA, USA) were used in all *in vitro* experiments. In addition, a non-cancerous MCF-10A (human mammary gland epithelial cells) were used as a normal breast epithelial model. MCF-7 cells were maintained in DMEM medium (GE Healthcare, Piscataway, NJ, USA), MDA-MB-231 in RPM1 1640 medium (Biosera, Kansas City, MO, USA), and MCF-10A in DMEM F12 medium (Biosera, Kansas City, MO, USA) + suppl. insulin, EGF- epithelial growth factor and HC-hydrocortisone (all Sigma, Steinheim, Germany). The 10% FBS (Fetal bovine serum, Gibco) and antibiotic/antimycotic solution (1× HyClone™; GE Healthcare, Chicago, IL, USA) were used to supplement culture media. Basic cultivation conditions were a 5% CO_2_-containing atmosphere, humidified air, and 37°C temperature. A trypan blue exclusion test was used to estimate the viability of used cells (≥95%). For the flow cytometry experiments, the MCF-7 (3 × 10^5^) and MDA-MB-231 (1 × 10^5^) cells were seeded in Petri dishes. After 24 h seeding and initial colony growth in a complete cultivation medium, the cells were treated with the SPGE (Calendula, Nová Ľubovňa, Slovakia) for 24, 48, and 72 h before analysis (Kubatka et al., 2019; Kubatka et al., 2020a; Kubatka et al., 2020b).

### 2.9 Cytotoxicity assay

The cytotoxic effects of SPGE and propylene glycol (concentration range 10–1,000 μg/mL) on MCF-7, MDA-MB-231, and MCF-10A cells were analyzed by resazurin metabolic assay. The 10 µL of the final concentration of 40 µM resazurin solution (Sigma) was added to each well after 72 h of incubation with SPGE and propylene glycol. After 1 h metabolization, the fluorescent product resorufin was analyzed and fluorescence was measured by the automated Cytation^TM^ 3 cell imaging multi-mode reader (Biotek, Winooski, VT, USA) at 560 nm excitation/590 nm emission filter set. The results were expressed as a fold of the control in which the fluorescence of the control wells was taken as 100%. The numbers of experimental repeats were set (n = 3) (Kubatka et al., 2019; Kubatka et al., 2020a; Kubatka et al., 2020b).

### 2.10 5-Bromo-20-deoxyuridine (BrdU) cell proliferation assay

Cell proliferation was analyzed by the BrdU incorporation ELISA assay following the manufacturing instructions (Roche Diagnostics GmbH, Mannheim, Germany). The incorporation of BrdU into genomic DNA during cell growth was quantified and analyzed after SPGE extract treatment (10–1,000 μg/mL) in our experiments. After the substrate degradation, the color intensity changes were read at 450 nm (reference wavelength: 690 nm) by Cytation^TM^ 3 Cell Imaging Multi-Mode Reader (Biotec). The numbers of experimental repeats were set (*n* = 3). Based on Resazurine and BrdU assays, the final IC_50_ was calculated and determined: 160 μg/mL (MCF-7 cells) or 130 μg/mL (MDA-MB-231 cells) (Kubatka et al., 2019; Kubatka et al., 2020a; Kubatka et al., 2020b).

### 2.11 Flow cytometry analyses protocol

The floating and adherent cells (MCF-7 or MDA-MB-231) were harvested and mixed for every sample after 24, 48, and 72 h following the treatment (SPGE extract final dilutions 160 or 130 μg/mL). Then, the samples were centrifuged, washed in PBS, and again resuspended in PBS according to the following staining protocol. For intracellular staining, cells were fixated by 4% paraformaldehyde (15 min), followed by washing and permeabilization with 0.5% TWEEN for 15 min at room temperature. The samples were divided for particular staining as is listed in the table below. Cells were stained for 15–30 min in the dark at room temperature. After a washing step (PBS), samples were resuspended in PBS, and the FACSCalibur flow cytometer (Becton Dickinson, San Jose, CA, USA) was used for fluorescence analyses (Kubatka et al., 2019; Kubatka et al., 2020a; Kubatka et al., 2020b).

A list of flow cytometry analyses and staining.

**Table:** 

Analyses	Staining solution	Company
Cell cycle *	Triton X-100 10% solution ribonuclease A 0.5 mg/mL propidium iodide–PI 0.025 mg/mL In 500 µL PBS	Sigma-Aldrich, Steinheim, Germany
Apoptosis	Annexin V-Alexa Fluor 647 1:100 (cat. no. A23204)	Thermo Scientific, Rockford, IL, USA
	PI (5 mg/mL) 1:500	Sigma-Aldrich
Mitochondrial membrane potential	TMRE (tetramethylrhodamine ethyl ester per chlorate) final conc. 0.1 µM	Molecular Probes, Eugene, OR, USA
Caspase activation	Cleaved caspase-3 rabbit mAb PE conjugate 1:100 (#9978)	Cell Signaling, Danvers, MA, USA
	Cleaved caspase-7 rabbit mAb PE conjugate 1:100 (#42542)	
Proteins analysis	Cleaved PARP rabbit mAb PE conjugate 1:100 (#8978)	
	Bcl-2 mouse mAb PE conjugated 1:100 (#26295)	
	Phospho-Bcl-2 (Ser 70) rabbit mAb Alexa Fluor 488 conjugate 1:200 (#2834)	

*After harvesting, the cold 70% ethanol was used to fix cell suspension and samples were kept at −20°C overnight.

### 2.12 The examinations of plant secondary metabolites in salvia extract

The propylene glycol extract of *S. officinalis* L. (Lamiaceae) herb solvent (Batch No. S-02-01-11-10-21; Calendula a.s., Nová Ľubovňa, Slovakia) was used for the following studies. The extract was produced in an extraction reactor by extracting haulm of *S.officinalis* in the extraction agent propylene glycol at a temperature of 80°C. After cooling, the mixture was filtered and the liquid extract was packed in shipping containers. Analytical HPLC measurements were obtained on an Agilent 1260 chromatographic system (1260 Vial sampler G7129A, 1260 Quat Pump G7111B, 1260 MCT G7116A, 1260 DAD HSG7117C, Agilent Technologies, Waldbronn, Germany) with MS AB SCIEX Triple Quad 3500 system (Framingham, USA). InfinityLab Poroshell 120EC-C18 (4.6 × 100 mm, 2.7 μm) column with InfinityLab Poroshell 120EC-C18 (4.6 × 5 mm, 2.7 μm) guard column were used, with a gradient elution of A: MeOH with 0.1% HCOOH (v/v) and 1 mM HCOONH_4_, and B: water with 0.1% HCOOH (v/v) and 1 mM HCOONH4; A:B 0 min 10:90 (v/v), in 36th min 100% A, in 50th min 100% A. The flow rate was 0.3 mL/min, column block temperature was 30°C. MS conditions: curtain gas N_2_ 25 L/min, temperature 450°C, gas 1 50 L/min, gas 2 40 L/min, ion spray voltage −4500 V, scan mode *m/z* 50-1,000, scan rate 1,000 Da/s, solvent delay time 4 min compounds were identified by comparison of retention times, UV, and MS profile with standards. The quantification was carried out by using calibration curves constructed based on measurements of corresponding standards. Analytical GC/MS analysis was carried out the same as described previously (Kubatka et al., 2019; Kubatka et al., 2020a; Kubatka et al., 2020b).

### 2.13 Statistical analyses

Data from rodent studies are shown as mean ± SEM. The one-way analysis of variance (ANOVA), Student’s t-test, and Mann–Whitney test were used as statistical methods in data analysis. The tumor volumes were counted by the formula: V = π × (S1)2 × S2/12 (S_1_, S_2_ are tumor diameters; S_1_ < S_2_). Data from *in vitro* studies are shown as mean ± SD and were analyzed by ANOVA test followed by the Bonferroni multiple comparisons test. In addition, we used ANOVA and Student–Newman–Keuls multiple comparison tests in the fluorescence assay analysis (Kubatka et al., 2019; Kubatka et al., 2020a; Kubatka et al., 2020b). Differences between groups were considered significant when *p* ≤ 0.05. In the mentioned data analyses, GraphPad Prism Comparison Software (version 5.01, La Jolla, CA, USA) was used.

## 3 Results

### 3.1 Evaluation of secondary metabolites in *S*. *officinalis* propylene glycol extract

The propylene glycol extract of *S. officinalis* was analyzed using CS-MS and LC-MS techniques. As expected, the GC-MS analysis did not reveal substantial amounts of volatile compounds in such type of extract (data not shown).

**FIGURE 1:**
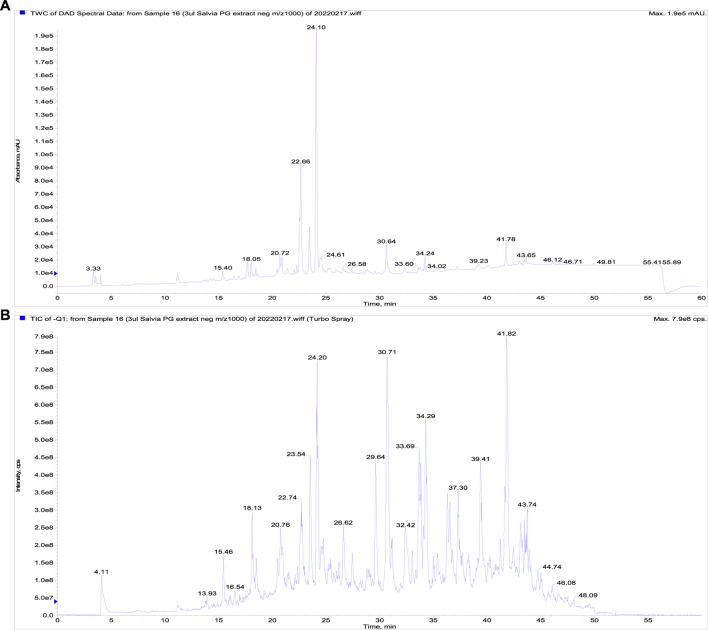
The LC-DAD-analysis of the propylene glycol extract of *S. officinalis*. **(A)** The UV signal showing the presence of phenolics, **(B)** a total ion current chromatogram (fast polarity switching mode).

The LC-DAD-MS analysis confirmed the presence of phenolics represented by rosmarinic, protocatechuic, and salicylic acids, furthermore, the presence of triterpenes ursolic and acids was proved (Figure 1). The metabolites were identified based on analysis of UV spectra and mass spectra and comparison of retention times with corresponding standards. Furthermore, we quantified these non-volatile substances in extract (Table 1). As visible, only rosmarinic acid and triterpene acids were present in important amounts. Interestingly, we did not identify the substantive presence of flavonoids.

**TABLE 1:** The quantification of main secondary metabolites identified in *S. officinalis* propylene glycol extract.

Compound	Retention time (min)	The amount in the extract [μg/mL] (RSD)
Rosmarinic acid	24.1	1,402 (0.6%)
Protocatechuic acid	12.4	4 (3.4%)
Salicylic acid	25.14	2 (0.38%)
Oleanolic acid	43.7	374 (10.8%)
Ursolic acid	43.6	650 (8.9%)

### 3.2 Allograft 4T1 model

**FIGURE 2:**
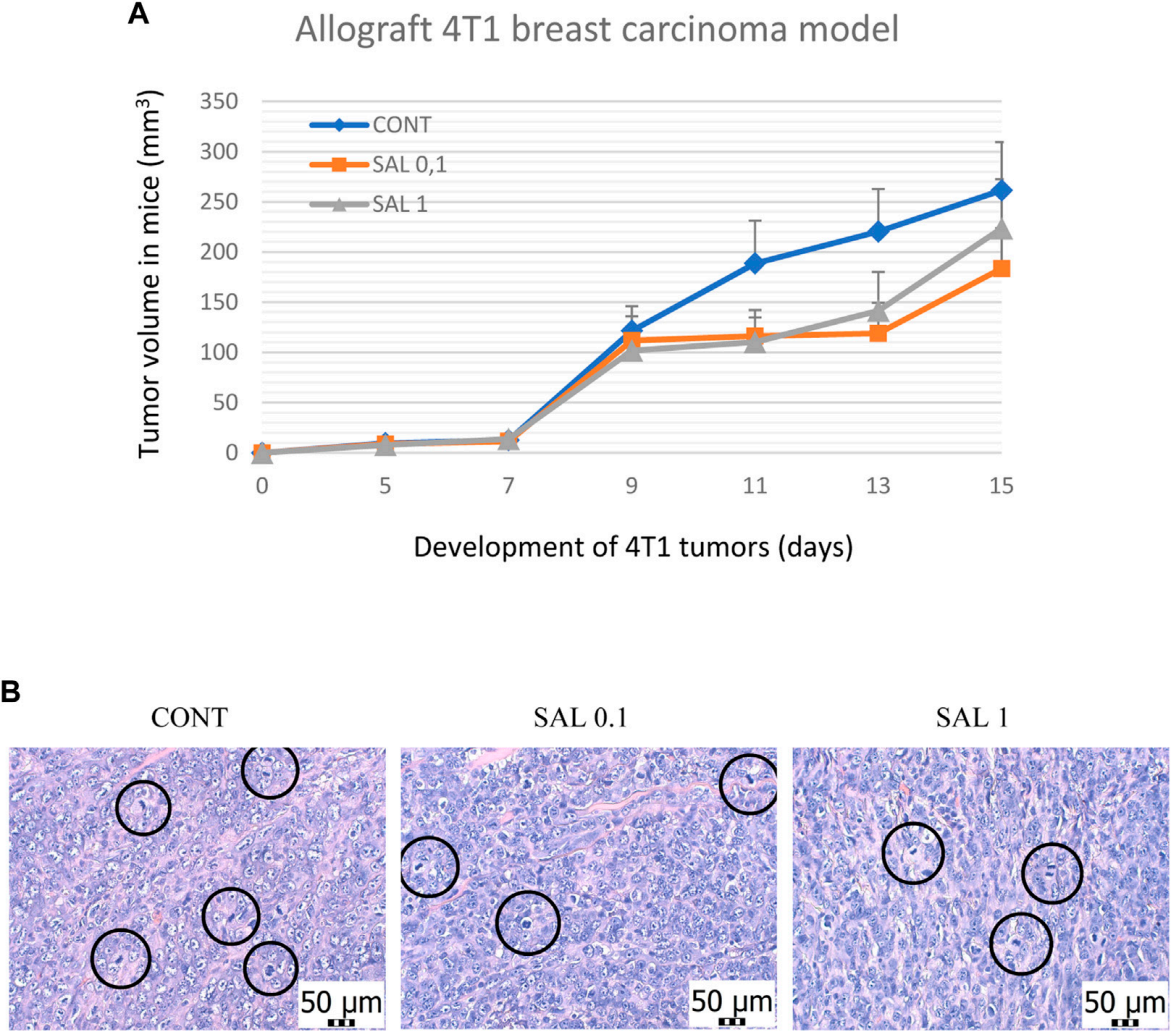
Allograft 4T1 model in mice. **(A)** The volume of 4T1 mammary adenocarcinomas in mouse allograft model after *S.officinalis* treatment. **(B)** The mitotic activity index after the treatment with *S. officinalis* extract in 4T1 tumors in Balb/c mice. The mitotic figures are highlighted in circles; H&E staining; magnification ×400. CONT–control group, SAL 0.1 – a group with dietary applied salvia at a concentration of 1 g/kg in the diet, SAL 1 – a group with dietary applied salvia at a concentration of 10 g/kg in the diet. Data are shown as mean ± SEM.

*S. officinalis* apparently (but not significantly) decreased the volume of mouse 4T1 tumors in both treated groups when compared with untreated animals (Figure 2). Histopathological evaluation of cancer cells revealed a significant decline in mitotic activity index after salvia therapy by 37.5% (SAL 0.1) and 31.5% (SAL 1) vs. controls (*p* < 0.001) (Table 2; Figure 2). Evaluation of the necrosis ratio in the whole tumor area revealed a significant reduction by 46% (*p* < 0.05) after a higher salvia dose in comparison with untreated (control) mice (Table 2).

**TABLE 2:** Histopathological evaluation of 4T1 tumor specimens after *S. officinalis* therapy in Balb/c mice.

Parameter	CONT	SAL 0.1	SAL 1
The ratio of necrosis in the whole tumor area	0.146 ± 0.023	0.168 ± 0.040	0.079 ± 0.015*
Mitotic activity index	30.25 ± 1.62	18.90 ± 1.00***	20.70 ± 2.13***

Data are shown as mean ± SEM. A significant difference, *p < 0.05, ***p < 0.001 vs. CONT.

### 3.3 Carcinogen-induced mammary carcinogenesis in rats

*S. officinalis* applied in higher doses showed a significant lengthening of tumor latency by 9 days (*p* < 0.05) vs. control animals without chemoprevention. Histopathological characteristics of salvia-treated rat tumor samples revealed a significant decline in the high-/low-grade carcinomas ratios after chemoprevention by 61% (*p* < 0.05) in the SAL 0.1 group and 58% (*p* < 0.05) in the SAL 1 group vs. controls. The tumor incidence, tumor frequency, and tumor volume observed in experimental groups did not reach the levels of significant differences (Table 3). Mixed papillary/cribriform carcinomas and cribriform/papillary carcinomas, also cribriform carcinomas and mixed cribriform/comedic carcinomas were the most occurring lesions in female rats. Sporadic lesions in rats were ductal carcinoma *in situ* (DCIS), papillary and tubular carcinomas, and cribriform/tubular and cribriform/papillary/comedo carcinomas.

**TABLE 3:** Effects of *S. officinalis* in chemoprevention BC model at the final week of the study.

Group	CONT	SAL 0.1	SAL 1
Tumor-bearing animals/all animals	18/25	20/25	20/25
Tumor incidence (%)	72	80	80
Tumor frequency per group	2.08 ± 0.48	2.28 ± 0.48	1.88 ± 0.43
Tumor latency (days)	73.71 ± 3.45	69.26 ± 2.72	82.16 ± 2.32*^,#^
Average tumor volume (cm^3^)	0.74 ± 0.20	0.84 ± 0.20	0.64 ± 0.22
High/low-grade carcinomas ratio	26/26 (=1.00)	16/41 (0.39)*	14/33 (0.42)*

CONT – control group, SAL 0.1 – a group with dietary applied salvia at a concentration of 1 g/kg in the diet, SAL 1 – a group with dietary applied salvia at a concentration of 10 g/kg in the diet. Data are shown as mean ± SEM. A significant difference, *p < 0.05, ^#^ *p* < 0.001 vs. SAL 0.1.

### 3.4 Rat tumor specimens–immunohistochemical analyses

#### 3.4.1 Parameters of apoptosis, proliferation, angiogenesis, and oxidative stress

**FIGURE 3:**
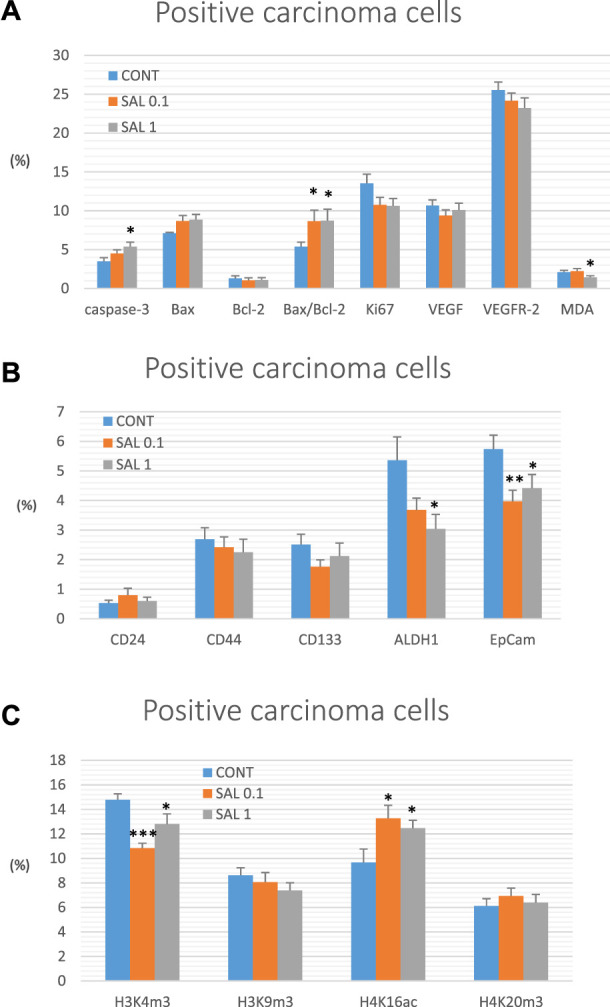
Immunohistochemical analyses of rat carcinoma cells *in vivo* after *S. officinalis* treatment. **(A)** Immunoexpression of cleaved caspase-3 (cytoplasmic), Bax, Bcl-2, Ki67, VEGFA, VEGFR-2, and MDA in rat tumor samples. **(B)** Immunoexpression of cancer stem cell markers in rat tumor samples. **(C)** Immunoexpression of H3K4m3, H3K9m3, H4K16ac, and H4K20m3 markers in rat tumor samples.

Salvia dose-dependently increased cytoplasmic expression of cleaved caspase-3 by 29% (*p* ˃ 0.05) and 54% (*p* < 0.05) compared to control tumors. Moreover, salvia elevated Bax/Bcl-2 expression ratio by 61% (SAL 0.1 group, *p* < 0.05) and 62.5% (SAL 1 group, *p* < 0.05) vs. the controls. Besides, salvia downregulated expression of Ki67 by 20.5% (SAL 0.1 group, *p* = 0.08) and 21.5% (SAL 1 group, *p* = 0.063) vs. control specimens. Finally, salvia at a higher dose decreased cellular levels of MDA by 30.5%, however, VEGF and VEGFR-2 as parameters of angiogenesis were not altered in treated groups vs. untreated tumor samples. The quantification of the above-mentioned parameters of apoptosis, proliferation, angiogenesis, and antioxidant activities of *S. officinalis* carcinoma cells *in vivo* is summarized in Figure 3A.

#### 3.4.2 Parameters of cancer stem cells

When compared to control tumor samples, analyses of CSCs markers in rat tumor specimens showed a dose-dependent decline in ALDH1 expression by 31.5% (*p* = 0.069) and 43.5% (*p* < 0.05) vs. controls and a dose-independent decline in the expression of EpCam by 31% (*p* < 0.01) and 23% (*p* < 0.05) vs. controls, respectively. Evaluation of other CSC markers (CD24, CD44, and CD133) did not reveal any significant difference when compared untreated and groups with *S. officinalis* (Figure 3B).

#### 3.4.3 Parameters of histones’ chemical modifications

Alterations in post-translation chemical modifications of histones H3 and H4 in rat carcinomas *in vivo* after the treatment with salvia are shown in Figure 3C. Compared to controls, salvia decreased H3K4m3 levels by 26.5% (SAL 0.1 group, *p* < 0.001) and 13.5% (SAL 1 group, *p* < 0.05), and elevated H4K16ac by 37.5% (SAL 0.1 group, *p* < 0.05) and 29% (SAL 1 group, *p* < 0.05). H3K9m3 and H4K20m3 levels were not significantly altered in *S. officinalis* groups vs. untreated group (Figure 3C).

Data are shown as mean ± SEM. A significant difference, *p < 0.05, **p < 0.01, ***p < 0.001 vs. CONT. The graphs show the protein expression, which is quantified as the average percentage of antigen-positive area in standard fields (0.5655 mm^2^) of hotspot areas within the tumor area. At least 60 pictures for each parameter were assessed.

**FIGURE 4:**
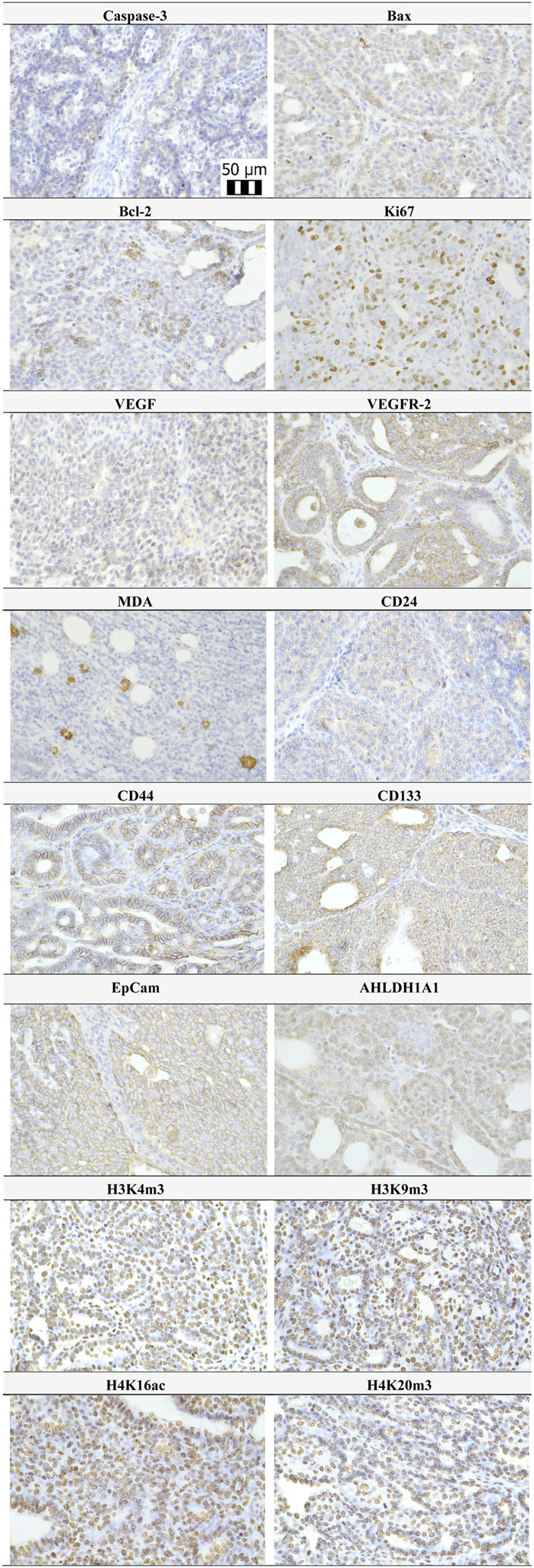
Representative pictures of caspase-3, Bax, Bcl-2, Ki67, VEGFA, VEGFR-2, MDA, CD24, CD44, ALDH1A1, EpCam, H3K4m3, H3K9m3, H4K20m3, and H4K16ac expressions gained from rat BC samples. For analysis, polyclonal caspase-3 antibody (Bioss, Woburn, USA), polyclonal Bax and Bcl-2 antibodies (Santa Cruz Biotechnology, Paso Robles, CA, USA), monoclonal Ki67 antibody (Dako, Glostrup, Denmark), monoclonal VEGFA and VEGFR-2 antibodies (Santa Cruz Biotechnology, Paso Robles, CA, USA), polyclonal CD24 antibody (GeneTex, Irvine, CA, USA), polyclonal CD44 antibody (Boster, Pleasanton, CA, USA), polyclonal ALDH1A1 antibody (ThermoFisher, Rockford, IL, USA), polyclonal MDA, EpCAM, H3K4m, H3K9m3, and H4K20m3 antibodies (Abcam, Cambridge, MA, USA), and monoclonal H4K16ac antibody (Abcam, Cambridge, MA, USA) were applied. The final microscope magnification of ×400 was used.

Representative expressions of cleaved caspase-3, Bax, Bcl-2, Ki67, VEGFA, VEGFR-2, MDA, CD24, CD44, CD133, ALDH1A1, EpCam, H3K4m3, H3K9m3, H4K16ac, and H4K20m3 in rat BC samples are summarized in Figure 4.

### 3.5 miRNA expression in rat tumor samples

**FIGURE 5:**
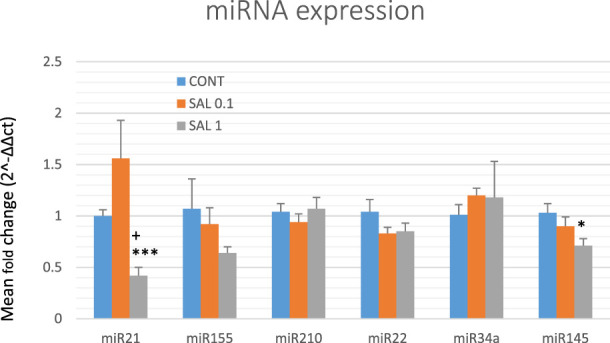
Relative miRNA expression of miR21, miR155, miR210, miR22, miR34a, and miR145 in rat BC specimens. MiR-191-5p was selected as the internal control miRNA to normalize the cDNA levels of the samples. Data are shown as mean ± SEM. A significant difference, *p < 0.05, ***p < 0.001 vs. CONT, ^*+*^ *p <* 0.05 vs. SAL 0.1.

Based on preclinical and clinical cancer research data, we chose six well-validated miRNAs to analyze the epigenetic mechanism of anticancer action of *S. officinalis* using tumor samples from the chemoprevention BC model (Figure 5). The higher dose of *S. officinalis* significantly downregulated the expression of miR21 by 58% (*p <* 0.001) and non-significantly downregulated miR155 expression by 40% (*p* = 0.194) vs. untreated cells. On the other hand, a higher dose of salvia significantly decreased miR145 expression by 31% (*p <* 0.05) in comparison with control tumor samples. Higher or lower doses of *S. officinalis* did not significantly alter the expressions of miR22, miR34a, and miR210 in comparison with untreated tumor specimens. In the comparison of SAL 0.1 vs. SAL 1 group, we revealed a significant decrease in miR21 expression by 73% (*p <* 0.05). Generally, a lower dose of *S. officinalis* was ineffective in expression modulations of selected miRNAs vs. the control tumor samples.

### 3.6 Promoters’ methylation status in tumor suppressor genes *in vivo*

**FIGURE 6:**
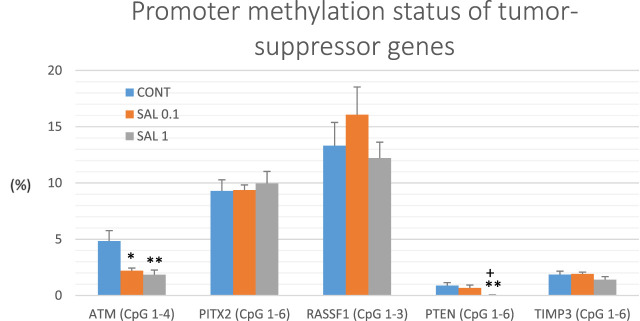
Promoter methylation status of *ATM*, *PITX2*, *RASSF1A*, *PTEN*, and *TIMP3* tumor-suppressor genes in rat BC specimens. The level of methylation was designated using all evaluated CpG isles of the above-mentioned promoters. The brackets indicate the number of evaluated isles. Data are shown as mean ± SEM. A significant difference, **p < 0.01, ***p < 0.001 vs. CONT group and ^*++*^ *p <* 0.01, ^*+++*^ *p <* 0.001 vs. SAL 0.1 group.

Gene promoter methylation analysis of the following tumor-suppressors was realized: ATM comprising four evaluated CpG sites (CpG 1–4), PITX2 (CpG 1–5), RASSF1 (CpG 1–3), PTEN (CpG 1–6), and TIMP3 (CpG 1–6) (Figure 6). We assessed twenty BC specimens for each experimental group in the rat chemoprevention study. *S. officinalis* dose-dependently declined total methylation of *ATM* promoter by 54.5% (*p* < 0.05) in lower doses and by 62% (*p* < 0.01) in higher doses vs. control samples. Evaluating the methylation of the *PTEN* promoter, we found a dose-dependent decrease of 25% (*p* = 0.55) and 96.5% (*p* < 0.01) in comparison with the untreated tumor samples. In addition, comparing the SAL 0.1 vs. SAL 1 group, we revealed a significant decrease of 95.5% (*p* < 0.05) in the methylation of the *PTEN* promoter. Besides, salvia at higher doses non-significantly reduced methylation of *TIMP3* promoter by 24% (*p* = 0.28) vs. controls. Analyzing two other tumor-suppressors–*PITX2* and *RASSF1*, treatment with *S. officinalis* did not cause changes in methylation of their promoter regions when compared to untreated (control) tumor samples (Figure 6).

### 3.7 Serum inflammatory cytokine levels in rats

**FIGURE 7:**
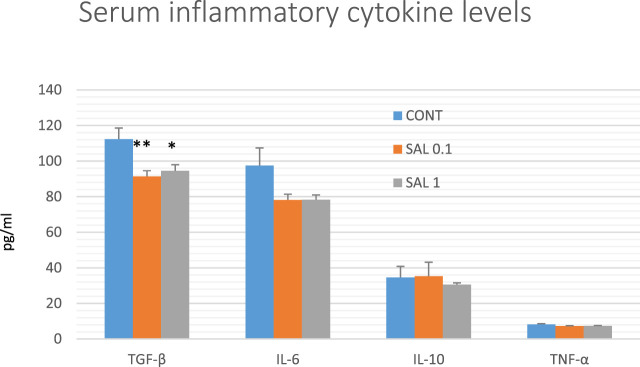
Serum levels of cytokines in rat chemoprevention study. Data are shown as mean ± SEM. TGF-β, transforming growth factor-beta; IL-6, Interleukin 6; IL-10, Interleukin 10; TNF-a, tumor necrosis factor-alpha. A significant difference, *p < 0.05, **p < 0.01 vs. CONT group.

The serum inflammatory cytokine levels in experimental groups are shown in Figure 7. Serum TGF-β levels were significantly reduced in both salvia groups by 18.5% (SAL 0.1, *p* < 0.01) and 16% (SAL 1, *p* < 0.05) in comparison with the control samples. A decrease in IL-6 and TNF-α levels in both treated groups showed a borderline significance compared to control groups (*p* < 0.10 in all comparisons). Levels of IL-10 were not changed in rat serum after the treatment with a diet medicated with salvia haulm.

### 3.8 Plasma metabolomic profile of tumor-bearing rats

**FIGURE 8:**
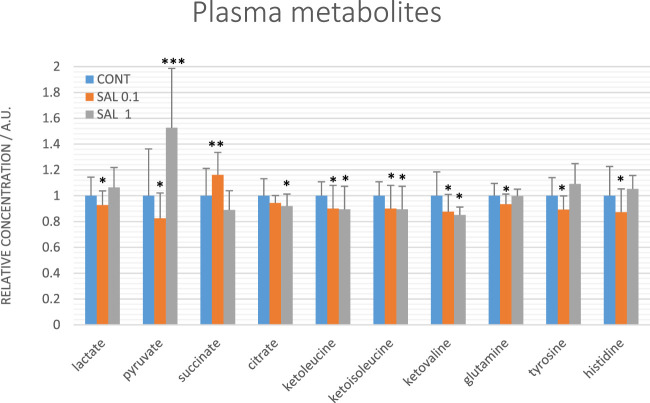
Relative concentrations of plasma metabolites in rats with mammary carcinomas. Data are shown as mean ± STD, normalized to the mean of controls set to 1. A significant difference, *p < 0.05, **p < 0.01, ***p < 0.001 vs. CONT.

Evaluating relative levels of circulating metabolites, we obtained significant changes between controls and salvia-treated animals in metabolites involved in energy metabolism: lactate, pyruvate, citrate, and succinate, further in tyrosine, glutamine, and histidine and a very similar course of changes common for all three ketoacids derived from BCAAs: ketoleucine, ketoisoleucine and ketovaline (Figure 8). The relative concentrations of 10 plasma metabolites were not significantly changed between controls (untreated) and salvia-treated groups; essential amino acids: leucine, isoleucine, valine, phenylalanine, threonine, tryptophan, ketone body representative 3-hydroxybutyrate, further alanine, acetate, and lysine.

### 3.9 Physiological *in vivo* effects of *S. officinalis*

In the final week of the experiment with rats, we found a significant increase in body weight by 22 g (SAL 0.1, *p* < 0.01) and 19 g (SAL 1, *p* < 0.05) when compared to controls (average weight - 266.4 g). In accordance with these data, we have revealed a significant rise in the food intake of treated rats by 2.6 g (SAL 0.1, *p* < 0.01) and 2.3 g (SAL 1, *p* < 0.01) vs. control animals (17.3 g of diet/rat/day). Prolonged application of salvia in the diet during the 13 weeks of the experiment was generally well tolerated by animals. The macroscopic organ pathologic alterations assessing gastritis, hepato-splenomegaly, liver steatosis, and hematopoietic disorders were not observed. Other side effects, e.g., abnormalities in vitality, mucosa, and hair were not seen in treated animals. Based on diet consumption, the mean salvia dose per rat/day was calculated as 19.85 mg in the SAL 0.1 group and 196.1 mg in the SAL 1.0 group. The mean salvia dose per mouse/day represented 2.97 mg (SAL 0.1) and 28.7 mg (SAL 1).

### 3.10 *In vitro* BC model

**FIGURE 9:**
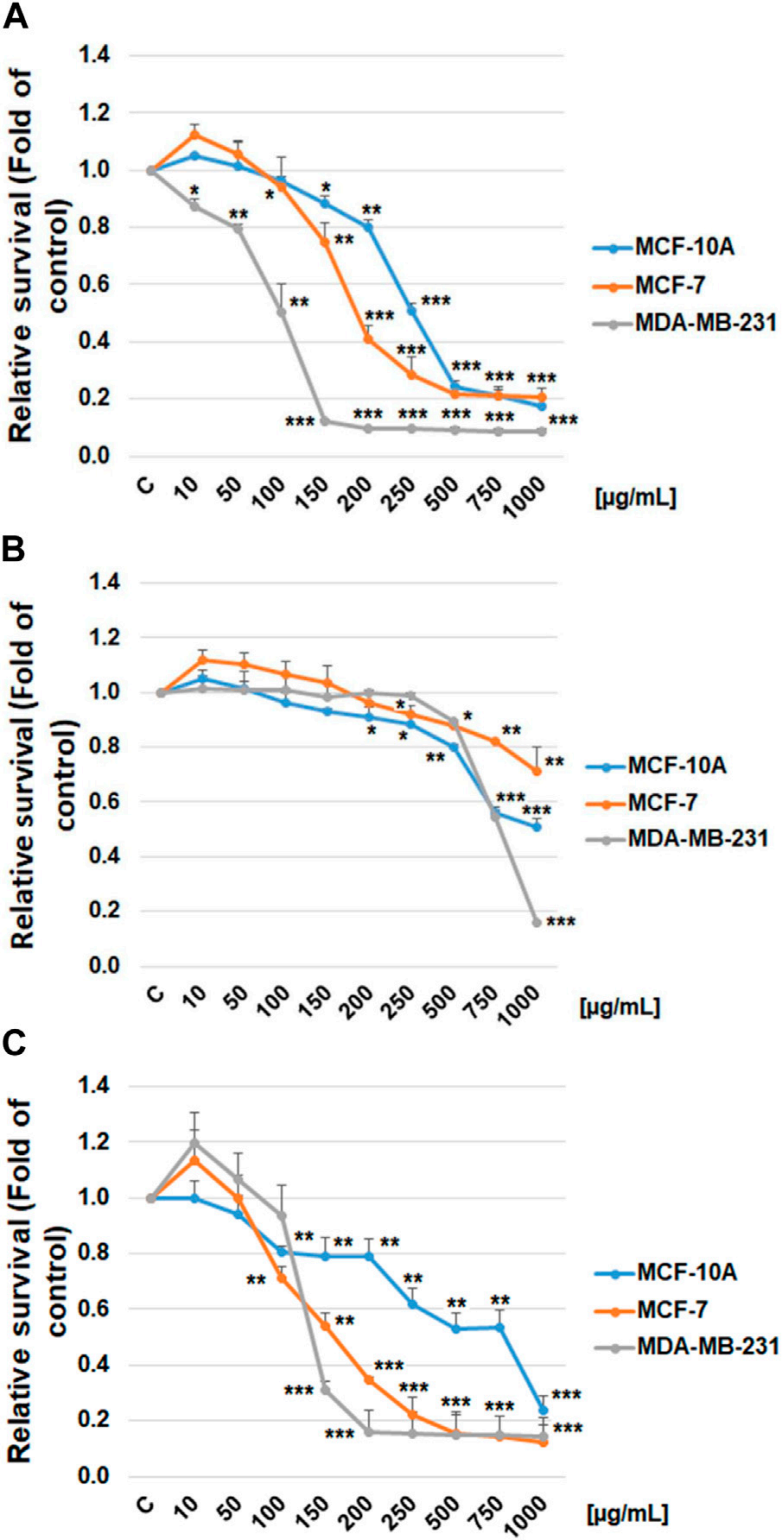
Relative survival of BC cells after salvia extract treatment. **(A)** Salvia propylene glycol extract (10–1,000 μg/mL) analyzed by resazurin metabolic assay, **(B)** propylene glycol dilutant analyzed by resazurin metabolic assay, **(C)** salvia extract (10–1,000 μg/mL) analyzed by BrdU assay. Data are shown as mean ± SD using three independent studies. Significant difference, *p < 0.05, **p < 0.01, ***p < 0.001 vs. CONT (untreated).

The possible cytotoxic and antiproliferative effect of propylene glycol salvia extract on MCF-7 and MDA-MB-231 BC lines and MCF-10A healthy breast gland epithelial cells were evaluated by the Resazurin metabolic assay and by BrdU incorporation assay. Data revealed that salvia extract reduced metabolic activity in both BC cell lines associated with reduced cell survival in a dose-dependent manner. Specifically, a stronger effect has been demonstrated in triple-negative MDA-MB-231 cells than MCF-7 cells. The metabolic activity of normal MCF-10A breast epithelial cells was affected in the same concentration less compared to both cancer cell lines (Figure 9A). Only high concentrations of 500 μg/mL and above showed the same toxicity. The propylene glycol (PG) analyses (Figure 9B) showed that dilutants at high concentrations of 500 μg/mL and above significantly reduced the metabolic activity of all tested cell lines.

The BrdU incorporation evaluation clearly showed that salvia extract dose-dependently suppressed the proliferation in both BC cell lines (Figure 9C). The MCF-10A breast epithelial cells revealed significantly less reduced cellular proliferation after salvia extract treatment compared to cancer cell lines. Comparing used cell lines, MDA-MB-231 cells showed in both tests greater sensitivity to salvia extract treatment, similarly MCF-7 cells. The IC50 values were calculated as 160 μg/mL (MCF-7), 130 μg/mL (MDA-MB-231), and 280 μg/mL (MCF-10A).

**FIGURE 10:**
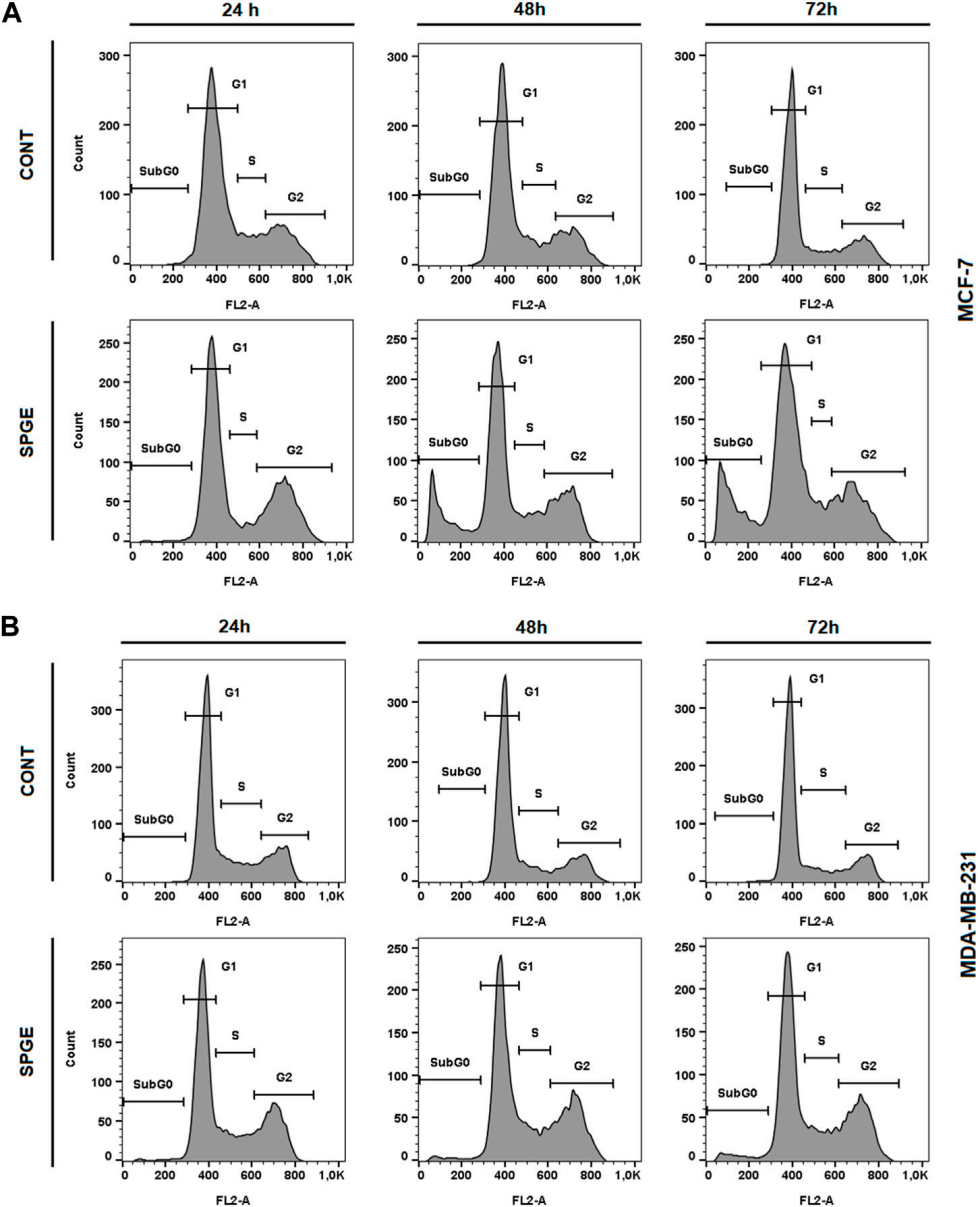
Sample diagrams of cell cycle distribution in BC cell lines after salvia extract treatment. **(A)** MCF-7 lines, **(B)** MDA-MB-231 lines; SPGE was applied at 160 or 130 μg/mL, resp.

The flow cytometry analysis of cell cycle progression, apoptosis occurrence, caspase activation, PARP cleavage, MMP changes, and Bcl-2 status after salvia extract treatment in MCF-7 and MDA-MB-231 lines were carried out after 24, 48, and 72 h. The cell cycle analyses clearly showed significant progression changes after salvia extract treatment (Table 4; Figure 10). In both cell lines, the G2/M cell cycle arrest occurred after 24 h of treatment, which maintained stable to 72 h but was stronger in MDA-MB-231 cells compared to MCF-7 cells. Moreover, the G2/M block increased in time in MDA-MB-231 cells while it weakened in MCF-7 cells. As a result of the cell cycle block, the apoptotic population with fractionated DNA (subG0/G1) occurred late at 48 h (MCF-7) or 72 h (MDA-MB-231) after salvia extract treatment. Moreover, the decrease of G1 phase arrested cells was recognized to be time-dependent. The subG0 population grew with time up to 10% maximum for 72 h in MCF7 cells and only up to 5% in MDA-MB cells. Importantly, a cell cycle block is observed in the G2/M phase for both lines, which is in line with the inhibition of proliferation since the cells in the block are waiting to see whether repair mechanisms at the DNA level will take place or not. The maximum 10% of the subG0 population, which we consider dead, is adequate for 72 h and the fact that the block is still visible for 48 h. In addition, since the block is more permanent on MDA-MB than MCF7, there is less subG0 population in MDA-MB cells.

**TABLE 4:** The cell cycle distribution of BC cell lines after salvia extract treatment.

MCF-7 cell line								
Time (h) treatment	24		48			72		
	CONT	SPGE	CONT		SPGE	CONT		SPGE
Sub-G_0_/G_1_	0.8 ± 0.1	2.3 ± 0.4	0.5 ± 0.1		9.8 ± 0.5*	0.6 ± 0.1		10.0 ± 0.4*
G_1_	64.1 ± 1.4	56.4 ± 0.1*	65.5 ± 0.3		57.4 ± 2.6*	66.8 ± 0.5		55.4 ± 3.6*
S	15.9 ± 2.5	9.9 ± 1.6*	13.5 ± 0.8		7.1 ± 0.6*	13.3 ± 0.3		9.7 ± 1.3*
G_2_/M	19.2 ± 1.1	31.5 ± 2.0**	20.5 ± 0.5		25.7 ± 2.8*	19.4 ± 0.9		25.0 ± 0.9*
MDA-MB-231 cell line								
Time (h) Treatment	24		48			72		
	CONT	SPGE	CONT	SPGE		CONT	SPGE	
Sub-G_0_/G_1_	0.6 ± 0.2	2.0 ± 0.8	0.9 ± 0.3	3.4 ± 0.3		1.0 ± 0.4	4.9 ± 0.6*	
G_1_	60.5 ± 0.6	52.4 ± 0.2*	67.0 ± 0.1	50.3 ± 1.0**		67.8 ± 2.1	50.4 ± 3.6**	
S	16.5 ± 1.1	18.3 ± 3.2	12.7 ± 0.4	15.5 ± 0.3		14.0 ± 1.3	12.5 ± 2.3	
G_2_/M	22.4 ± 1.4	27.3 ± 1.5*	19.4 ± 0.2	30.9 ± 0.4*		17.2 ± 0.4	32.4 ± 0.3**	

The cell cycle distribution in MCF-7 cells and MDA-MB-231 cells after salvia extract treatment (160 or 130 μg/mL, resp.) was assessed by flow cytometry. Data are shown as mean ± SD using three independent studies. Significant difference, *p < 0.05, **p < 0.01 vs. CONT.

We can assume that the G2/M block is related to DNA damage with activated DNA repair machinery or microtubule alteration contributed probably to mitotic catastrophe mediated by salvia extract. Further investigation is needed to clarify our assumption.

**FIGURE 11:**
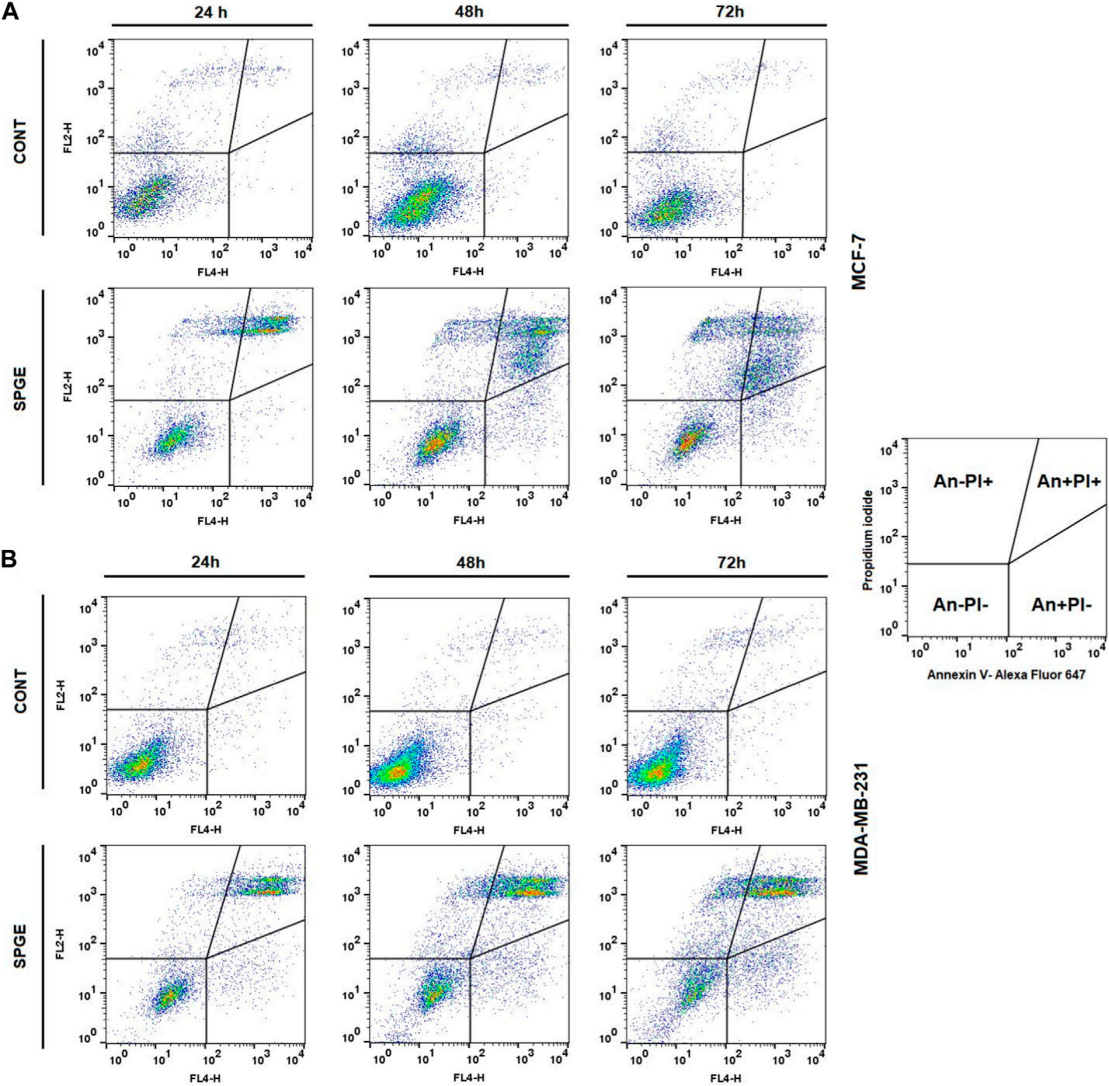
Sample diagrams of apoptotic cell diversification. **(A)** MCF-7 line, **(B)** MDA-MB-231 line; salvia extract was applied at 160 and 130 μg/mL, resp.

Following the cell cycle analyses, we focused on early and late events accompanied by cell death. The externalization of phosphatidyl serine (PS) on the outer cell membrane represents the early apoptotic marker. We demonstrated that salvia extract treatment in both BC cell lines led to a significant increase of PS externalization analyzed by Annexin V/PI staining at 24–72 h (Table 5; Figure 11). The result showed that salvia extract increased Annexin positivity in the population mainly in the late apoptotic state at 24–72 h of treatment. MDA-MB-231 cells showed a higher amount of late apoptotic cells and death cells (propidium iodide positive only) in all tested time points compared to MCF-7 cells. The delay in apoptosis occurrence is probably due to the G2/M cell cycle arrest.

**TABLE 5:** Flow cytometric analyses of apoptosis induction in BC cell lines after salvia extract treatment.

MCF-7 line						
Time (h) treatment	24		48		72	
	CONT	Salvia extract	CONT	Salvia extract	CONT	Salvia extract
An^−^/PI^−^	88.3 ± 4.2	68.0 ± 3.5*	90.8 ± 0.4	67.5 ± 3.8**	91.3 ± 0.9	47.1 ± 1.3**
An^+^/PI^−^	0.3 ± 0.1	0.8 ± 0.3	0.6 ± 0.2	3.6 ± 2.1	1.0 ± 0.4	7.2 ± 0.5*
An^+^/PI^+^	1.9 ± 0.8	21.7 ± 0.3**	1.5 ± 0.1	21.0 ± 0.6**	1.2 ± 0.2	29.5 ± 3.6**
An^−^/PI^+^	9.4 ± 3.2	9.6 ± 2.1	7.1 ± 0.1	7.9 ± 1.6	6.7 ± 1.5	16.2 ± 2.9*
MDA-MB-231 line						
Time (h) Treatment	24		48		72	
	CONT	Salvia extract	CONT	Salvia extract	CONT	Salvia extract
An^−^/PI^−^	89.6 ± 0.5	54.2 ± 4.8**	93.5 ± 1.3	47.0 ± 1.9**	92.5 ± 0.2	36.5 ± 3.8**
An^+^/PI^−^	1.9 ± 0.1	5.6 ± 0.4	1.3 ± 0.1	7.2 ± 0.6*	1.5 ± 0.4	15.2 ± 3.6*
An^+^/PI^+^	2.8 ± 0.2	25.8 ± 4.5**	1.7 ± 0.1	27.9 ± 2.0**	2.9 ± 0.6	35.1 ± 1.7**
An^−^/PI^+^	5.7 ± 0.8	14.5 ± 2.1*	3.4 ± 1.4	18.0 ± 3.9*	3.2 ± 0.8	13.3 ± 2.8*

An^−^/PI^−^ (lower left quadrant; non-apoptotic population), An^+^/PI^−^ (lower right quadrant; early apoptotic population), An^+^/PI^+^ (upper right quadrant; late apoptotic population), An^−^/PI^+^ (upper left quadrant; necrotic population). SPGE was applied at 160 μg/mL (MCF-7 cells) or 130 μg/mL (MDA-MD-231 cells). Data are based on three independent studies. Significant difference, *p < 0.05, **p < 0.01 vs. CONT.

**FIGURE 12:**
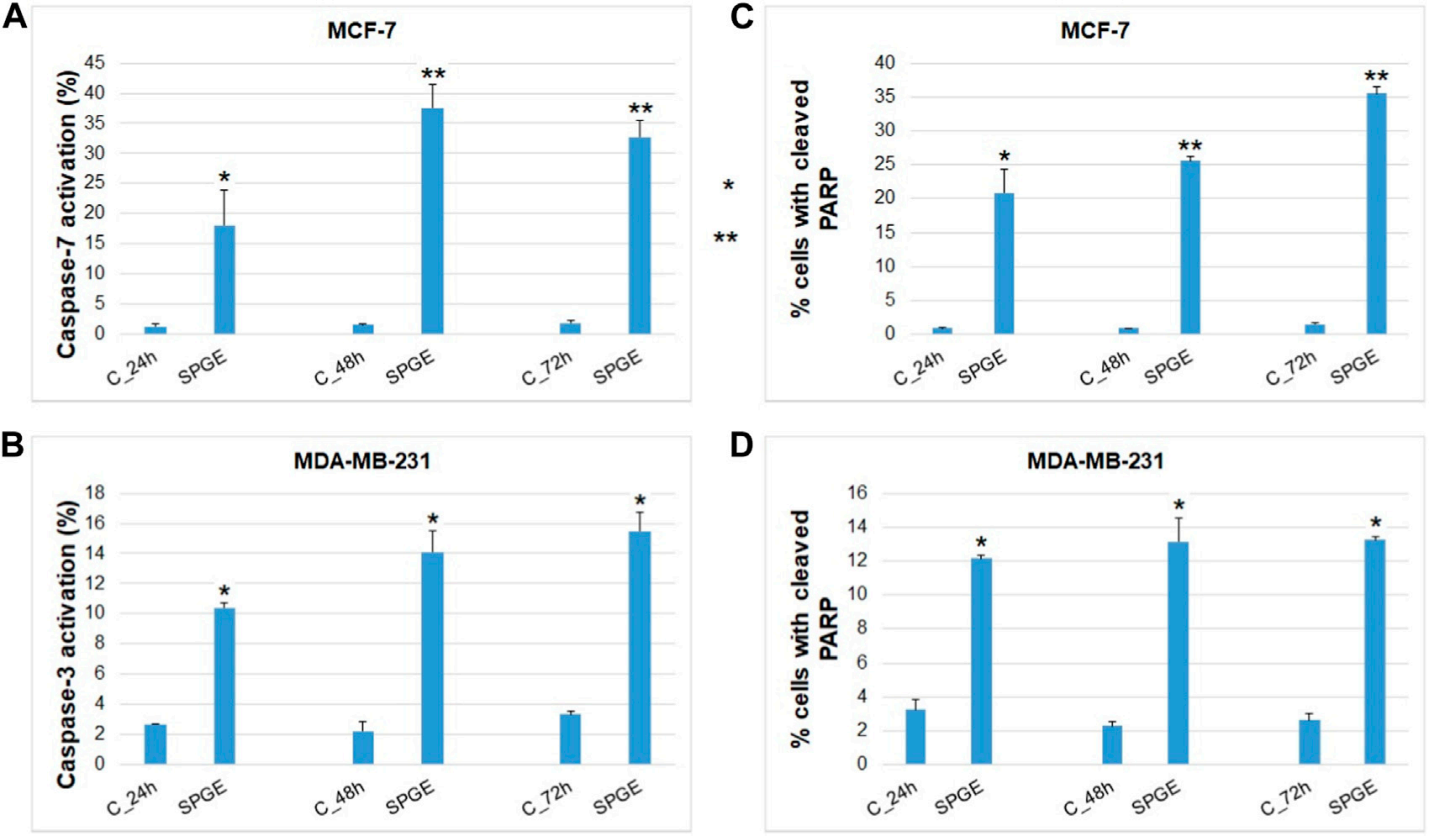
Effect of salvia extract on apoptosis parameters in BC cell lines analyzed by flow cytometry. **(A)** caspase-7 activation (MCF-7), **(B)** caspase-3 activation (MDA-MB-231), **(C, D)** PARP cleavage (MCF-7 and MDA-MB-231 cells). Salvia extract was applied at 160 and 130 μg/m resp. Data are based on three independent studies. Significant difference, **p < 0.01, ***p < 0.001 vs. CONT.

Moreover, we studied other mechanisms of salvia extract activity in MCF-7 and MDA-MB-231 lines representing key characteristics in the initiation and execution of apoptosis. As shown in Figures 12A–D, the intrinsic mitochondrial pathway of programmed cell death with caspase-dependent form was induced after SPGE application in both BC cell lines.

**FIGURE 13:**
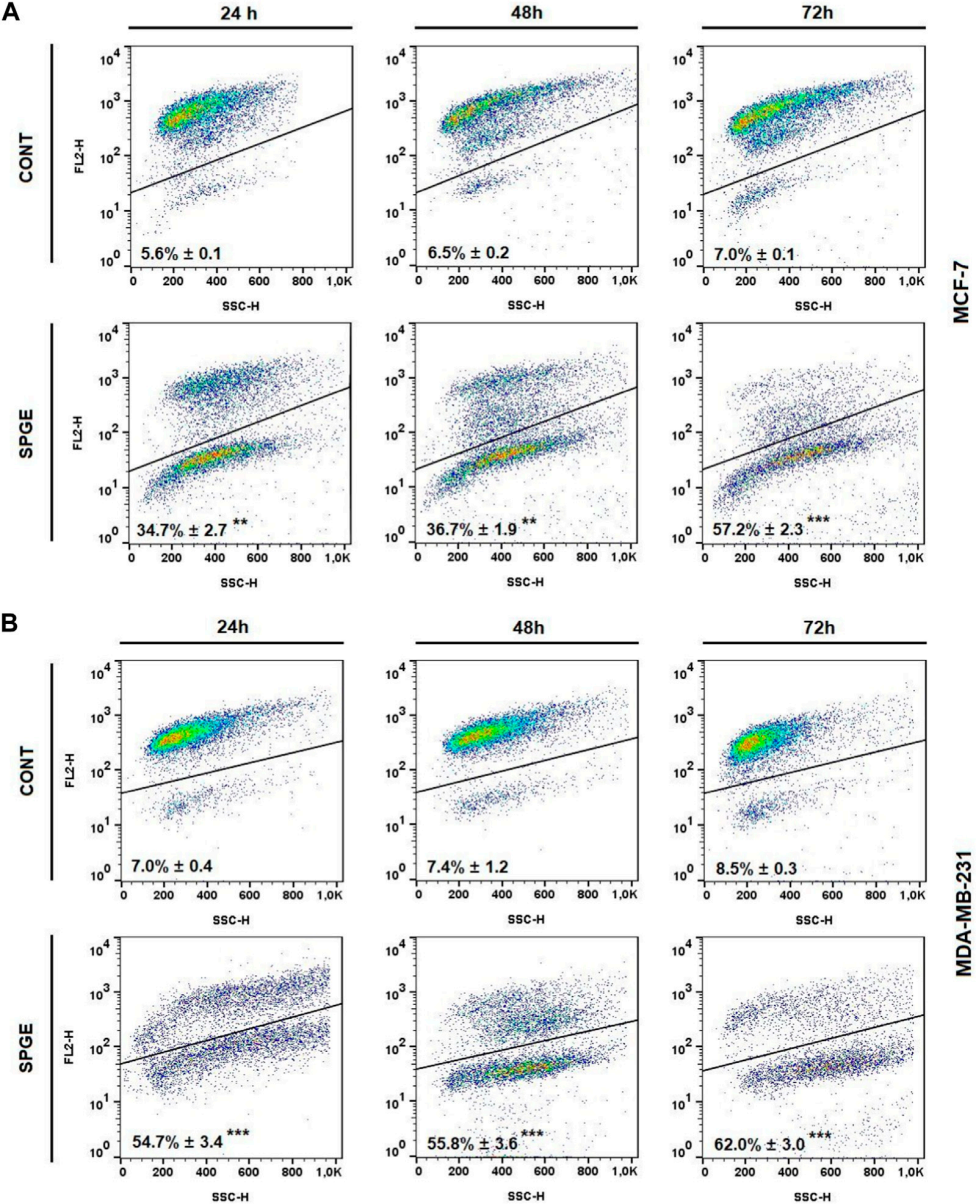
Mitochondrial membrane potential (MMP) in BC cell lines after salvia extract treatment. **(A)** MCF-7 cell line, **(B)** MDA-MB-231 cell line; salvia extract was applied at 160 and 130 μg/mL, resp. Data are based on three independent studies. Significant difference, **p < 0.01, ***p < 0.001 vs. CONT.

**FIGURE 14:**
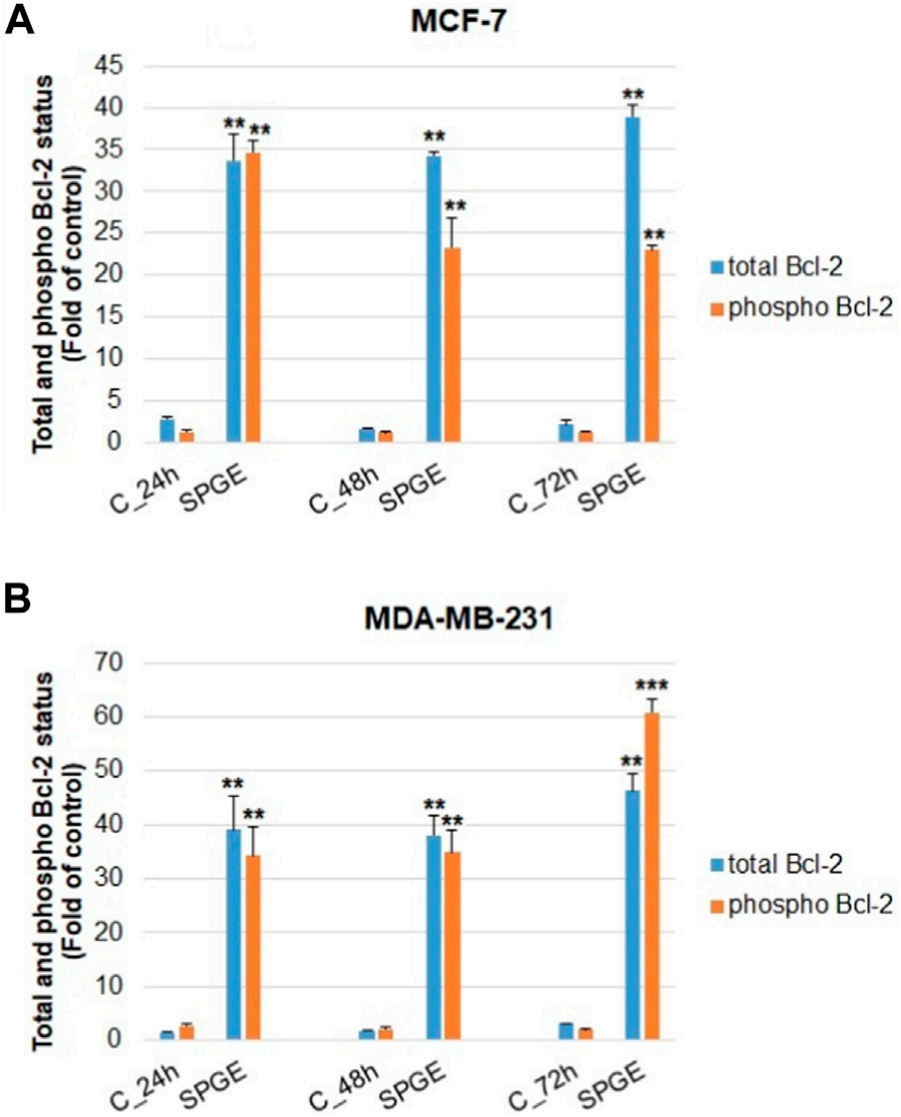
Flow cytometric evaluation of mitochondria-associated Bcl-2 protein in BC cell lines after salvia extract treatment. **(A)** Total Bcl-2, **(B)** phosphorylated Bcl-2; salvia extract was applied at 160 and 130 μg/mL, resp. Data are based on three independent studies. Significant difference, *p < 0.05, **p < 0.01, ***p < 0.001 vs. CONT.

The analyses of caspase-7 and caspase-3 activation (Figures 12A, B) confirmed a caspase-dependent mode of apoptosis in MCF-7 and MDA-MB-231 BC lines after salvia extract treatment in a time-dependent manner (24–72 h). Similarly, as execution caspases, cleaved PARP protein levels in both cell lines were increased after salvia extract treatment (Figures 12C, D), which is a clear marker of running apoptosis. In a comparison of both cell lines, the results showed that MCF-7 cells are more sensitive than MDA-MD-231 cells. Besides, the time-dependent decrease of mitochondrial membrane potential (MMP) in both BC cell lines was found after salvia extract treatment (Figure 13). The disruption of MMP initiates the mitochondria-localized changes and triggers apoptosis. The mitochondrial membrane proteins from the Bcl-2 family belong to the apoptosis-associated proteins that mediate pro- and anti-apoptotic signaling. The analyses showed that the anti-apoptotic protein Bcl-2 was released from mitochondria to cytosol in both BC cell lines in a time-dependent manner (24–72 h) after salvia extract treatment. The high phosphorylation status of Bcl-2 protein after salvia extract application indicates the deactivation of anti-apoptotic processes (Figure 14). Moreover, phosphorylation status changed time-dependently and reflected cell status based on cell cycle block and apoptosis occurrence. While phosphorylation of Bcl-2 decreased time-dependently in MCF-7, an increase in MDA-MB-231 occurred 72 h after treatment.

## 4 Discussion

Meta-analyses of clinical trials assessing regular consumption of plant foods and/or plant-derived molecules on the BC risk point to beneficial effects in the population (Farvid et al., 2021; Poorolajal et al., 2021). Other meta-analyses revealed that dietary management in BC patients based on vegetable and fibre intake provides useful lifestyle intervention against BC development (Wang et al., 2022). Despite constant progress in the advancement of anti-cancer strategies, the development of cancer resistance remains the main reason for BC-related mortality. In this regard, comprehensive preclinical research documented that targeting specific molecular pathways by phytochemicals to enhance therapeutic response by reversing the resistance and/or increasing sensitivity of cancer cells towards currently used therapeutic procedures could represent the perspective way in the management of BC (Liskova et al., 2021). To find novel medicinal plants with clinically beneficial anti-cancer activities, we evaluated the oncostatic potential of *S*. officinalis in therapeutic and preventive rodent models of BC and human cell BC lines.

The doses of salvia administered in the diet in both types of selected rodent models were based on our previous rich experience with the application of plant nutraceuticals in food (Kubatka et al., 2017b; Kubatka et al., 2019; Kubatka et al., 2020a; Kubatka et al., 2020b). The dose of salvia in the SAL 0.1 group (0.1% in the diet) is similar to the human dosing per day typical for the Mediterranean diet. Moreover, due to the supposed different pharmacokinetics and pharmacodynamics of phytochemicals present in salvia between humans and rodents, we used also a ten times higher dose, Data from this study showed significant anti-cancer activities of *S. officinalis* in rodent BC models. In the mouse 4T1 syngeneic study, a non-significant (but apparent) decline in tumor volume in mice after salvia treatment was associated with a significant reduction in necrosis ratio within the tumor sample and mitotic activity index. Our recent experiments using the same murine model and doses of dietary administered *Rhus coriaria* L. fruit peels, Cinnamomum zeylanicum L. bark, and *Thymus vulgaris* L. haulm showed significant therapeutic effects after comparison with control animals (Kubatka et al., 2019; Kubatka et al., 2020a; Kubatka et al., 2020b). The mentioned anti-cancer effects of plants used as medicinal plants or spices found in our laboratory were comparable with the data gained with conventional synthetic drugs analyzed using the same 4T1 BC murine model (Demečková et al., 2017; Solár et al., 2017; Grasselly et al., 2018). Even though the most sensitive parameter of experimental carcinogenesis, tumor frequency, was not changed, the rat BC model of primary chemoprevention in this study revealed a significant lengthening of tumor latency after higher salvia dose and significant shifted high-grade mammary carcinomas to low-grade lesions after application of both salvia doses when compared to controls. Simultaneously, no parameter was negatively affected by *S. officinalis,* which is a result demonstrating the chemopreventive effect of salvia in the BC model. Using the same rat model and similar doses of natural substances, significant chemopreventive effects and improvement in histopathological characteristics of tumors after an application of dark fruits, various medicinal plants, or spices were found in our laboratory (Kubatka et al., 2015; Kubatka et al., 2016a; Kubatka et al., 2016b; Kubatka et al., 2017a; Kubatka et al., 2017b; Kubatka et al., 2019; Kubatka et al., 2020a; Kubatka et al., 2020b) and by other authors (Singletary et al., 1996; Ravoori et al., 2012; Jeyabalan et al., 2014; Bishayee et al., 2016).

Based on our LC-DAD-MS analysis, the dominant metabolites present in SPGE are phenolic derivatives represented by rosmarinic acid and triterpenes represented by ursolic and oleanolic acids. Organic Anion Transporters are the major transporters that may modulate the pharmacokinetics of rosmarinic acid (Kang et al., 2021). In addition, intestinal transport of rosmarinic acid was mainly realized by conjugated forms with glucuronic acid or sulfate across Caco-2 cells through passive diffusion (Woottisin et al., 2022). PXR-ABC drug transporters are responsible for the membrane transport of CYP-mediated ursolic acid *in vivo* and *in vitro* (Jinhua et al., 2020). The research indicates that oleanolic acid monoglucoside is transported through the cell membrane by an active, carrier-mediated mechanism; on the other hand, the transport of monoglucuronide is a passive, carrier-dependent process (Szakiel and Janiszowska, 2002). These compounds could be assigned as responsible for the observed effects of salvia extract in both animal and cell models. Anticancer activities of rosmarinic acid were described and reviewed repeatedly, including effects in models based on BC cell lines and animals (Sharma et al., 2022; Zhao et al., 2022). As described previously, the bioavailability of triterpenes was analyzed by numerous studies (Furtado et al., 2017) and several studies also analyzed the bioavailability of phenolic acids, such as rosmarinic acid (Guan et al., 2022). However, the direct bioavailability of rosmarinic acid is relatively low and metabolic degradation producing smaller compounds like ferulic, caffeic, and 3,4-dihydroxyphenyl lactic acids (Hitl et al., 2021) was described. Numerous research data concluded higher tumor-suppressive effects of natural phytochemical cocktails occurring in whole foods intake when compared to single phytochemicals (Kapinova et al., 2017). It seems logical that synchronous targeting of multiple signaling molecules within the cell associated with carcinogenesis *via* numerous bioactive substances present in medicinal plants may represent a more effective clinical approach in cancer management when compared to monotherapy. As far as we know, there is no data on the adverse effects associated with long-term *S. officinalis* administration during its usage in humans for many centuries (Tildesley et al., 2005). The usage of *S.officinalis* in common doses within the Meditteranean diet is very safe; however, there might be undesirable side effects in excessive amounts, which can be attributed to the high content of thujone (Walch et al., 2011). However, the application of data gained from preclinical research to cancer patients is a very difficult task for oncologists. In this regard, translational research must solve several important issues crucial for the use of medicinal plants in clinical practice: (1) pharmacokinetic parameters of individual phytochemicals/metabolites; (2) sufficient levels of phytochemicals/metabolites in human plasma (promising method seems to be nanotechnology and advanced drug formulations); (3) effective and safe dosing; (4) combined application with the conventional drugs to increase the effects of anticancer therapy; (5) re-sensitizing of the chemotherapy/radiotherapy-resistant cancers; (6) impact of phytochemicals on cell signaling pathways to affect specific stages of carcinogenesis such as cell invasion/metastasis or disease relapse; (7) understanding the individual characteristics of patients within the multi-omics data integration for a more preventive, predictive, and personalized medical approach using plant medicinal foods.

Oncostatic characteristics of plant medicinal foods are well evidenced to be based on usual mechanisms of action such as programmed cell death, effects on cell cycle/proliferation and angiogenesis, or antioxidant activities that are induced by specific mixtures of secondary metabolites present in specific plants. Apoptosis represents a strictly organized process that is controlled by several cellular proteins and signaling pathways (Abotaleb et al., 2018; Abadi et al., 2022). Pro-apoptotic proteins are classified into caspases and Bcl2 family molecules. Caspases as cysteine proteases play a crucial role in apoptosis initiation. Caspases are usually present in the cell as inactive heterodimers - procaspases. Regarding the role of Bcl2 proteins in apoptosis, under stress cellular conditions these molecules are overexpressed to interact and sequester the pro-apoptotic Bax and Bak proteins and thus preserve the integrity of the mitochondrial membrane. Apoptosis can be mediated *via* two signaling pathways, intrinsic and/or extrinsic. The intrinsic pathway is modulated by the mentioned molecules of the Bcl2 family, whereas increased levels of the Bax/Bcl2 heterodimer activate the caspase-3 and subsequently initiate apoptotic changes in cancer cells (Abotaleb et al., 2018). Numerous research data described significant regulatory effects of phytochemicals in Bax/Bcl-2/caspase-3 signaling on cancer (Khan et al., 2021; Liu et al., 2021; Salamatullah et al., 2021). This study documented a significant rise in the Bax/Bcl-2 ratio in rat BC specimens *in vivo* after the treatment with both salvia doses. This result correlated with the upregulated expression of cleaved caspase-3 in the group with a higher salvia dose. We found similar data also in our previous experiments using the same rat model in which we demonstrated a significant correlation between caspase-3 expression and an increase in Bax/Bcl-2 ratio after the chemoprevention with dark fruit, sumac peels, oregano haulm, clove buds, and cinnamon bark (Kubatka et al., 2016b; Kubatka et al., 2017a; Kubatka et al., 2017b; Kubatka et al., 2020a; Kubatka et al., 2020b). The pro-apoptotic activities of salvia extract in this study were also assessed in our parallel evaluation using MCF-7 and MDA-MB-231 BC cells *in vitro*. In this regard, we analyzed early and late apoptosis mechanisms. The data indicated that SPGE treatment damaged the mitochondria membrane’s normal state in early events accompanied by release and a decrease in Bcl-2 expression. Alongside the increased Bcl-2 phosphorylation, the loss of MMP occurred very early. It is known that mitochondrial dysfunctions induce apoptosis and loss of MMP or release of anti- and pro-apoptotic mitochondrial proteins (Bcl-2, Bad, Bax, cytochrome c) may be the first feature of intrinsic cell death (Wang and Youle, 2009). Furthermore, we observed late apoptosis markers, i.e., activated caspase-3 and -7, and cleavaged PARP protein after salvia extract treatment in both BC cell lines. The similar apoptotic manifestation was found also after treatment with different natural extracts performed in our laboratory (clove buds, thyme, cinnamon, or sumac) (Kubatka et al., 2017b; Kubatka et al., 2019; Kubatka et al., 2020a; Kubatka et al., 2020b).

Isolated phytochemicals or medicinal plants demonstrate a significant impact on the division of BC cells (Adams et al., 2010; Han et al., 2021; Shehata et al., 2021). In this regard, phytochemicals target numerous signaling pathways and mechanisms such as PI3 kinase, Nrf2, COX-2, NF-κB, poly-ADP-ribosylation, Plk1, STAT3, Hedgehog, Wnt, or epigenetic changes that are included in the modulations of cell cycle and proliferation (Wang et al., 2012). The immunohistochemical analysis of the expression of nuclear protein Ki67, a highly valid proliferation marker, revealed a boundary-significant dose-dependent decline in rat tumor samples of both treated groups by 20.5% and 21.5% vs. control tumors. Moreover, salvia in both doses markedly decreased the mitotic activity index and necrosis ratio (only in higher doses) in 4T1 tumors. In addition, using resazurin viability assay and BrdU analysis, we found similar data as in rodent studies *in vivo*. SPGE declined the cell viability, metabolic activity, and proliferation in both human BC cell lines. The blocking of the cell cycle by salvia extract was confirmed by the G2/M phase arrest and subG_0_/G1 sub-population increase in MCF-7 and MDA-MB-231 BC cells. The different effect of *S. officinalis* on ER+ (MCF-7) and TNBC (MDA-MB-231) cells *in vitro* was partially manifested. Triple-negative BC cells were more resistant than ER+ BC cells, and we found a slightly stronger G2/M block, which is related to a time shift in other monitored parameters. It is well known that persistent or transient G2/M arrest is a prerequisite step for apoptosis and proliferation inhibition elicited by varying doses of substances with anticancer potential (Shu et al., 1997). Targeting the S and G2 checkpoints is also attractive for cancer therapy because the loss of G1 checkpoint control is a common feature of cancer cells, making them more reliant on the S and G2 checkpoints to prevent DNA damage–triggered cell death.

However, our previous studies analyzing the anticancer activities of Chlorella pyrenoidosa L. and Thymus vulgaris L. haulm showed clear discrepancies in markers of cell proliferation between *in vitro* and animal cancer models (Kubatka et al., 2015; Kubatka et al., 2019). The reason for such conflicting results and different therapeutic outcomes using mentioned medicinal plants is the heterogeneous genotype/phenotype of cancer cells in human lines and NMU-induced rat adenocarcinomas.

Several hypotheses have been described regarding the key role of tumor angiogenesis in tumor development and metastasis, while preventive/therapeutic inhibition of angiogenesis may represent an outstanding clinical approach in the management of carcinogenesis. Oncology research described numerous candidate molecules including phytochemicals that effectively affect the neovascularization linked with cell signaling molecules such as VEGF, FGF, EGF, or HGF (Niland and Eble, 2019). In addition, multiple factors such as tyrosine kinase receptors, integrins, and neuropilins interact with VEGF/VEGFR signaling and induce cancer initiation. Numerous bioactive plant-derived molecules can bind with VEGF-stimulating factors and suppress them, thereby inhibiting cancer development (Parveen et al., 2019). Salvia in our study did not modulate either VEGF nor VEGFR-2 expression levels in rat tumor samples. Using the same model, sumac in our previous study showed the same result. Conversely, in our two other recent animal studies, we observed a downregulation in VEGFR-2 expression after thyme (Kubatka et al., 2019) and VEGF expression after cinnamon treatment in rat tumor tissues (Kubatka et al., 2020a). Significant anti-angiogenic effects of natural cocktails of phytochemicals present in medicinal plants were demonstrated in our older rodent cancer studies (Kubatka et al., 2016b; Kubatka et al., 2017a; Kubatka et al., 2017b). However, comprehensive analyses on the effect of single phytochemicals/plant foods on the mechanisms of anti-cancer action and complex cell signaling within the process of angiogenesis and vasculogenesis are strongly warranted.

*S. officinalis*, through a high flavonoid and phenolic acid content, demonstrates effective anti-inflammatory and antioxidant characteristics (Kolac et al., 2017). Chronic, non-physiologic inflammation is associated with the pathophysiology of numerous diseases *via* inducing oxidative stress and consequent cellular damage. We have found a significant decline in serum TGF-β cytokine levels and borderline significant decreases in serum IL-6 and TNF-α cytokine levels. TGF-β is a multifunctional cytokine that behaves as a tumor promoter or tumor suppressor in dependence on a cellular stage and content. Scientific data showed that TGF-β is actively produced by tumor cells (mainly in the later stages of carcinogenesis) and contributes to cell growth, migration, and metastatic spread, and in addition, represses host-tumor immune responses (Kim et al., 2021). Similarly, TNF-α stimulates BC growth through the positive feedback loop of TNFR1/NF-κB (and/or p38)/p-STAT3/HBXIP/TNFR1 (Cai et al., 2017). Despite the knowledge that TNF-α supports apoptosis, the knock-out of this cytokine can upregulate different pro-apoptotic signaling in certain BC subtypes that regulate specific molecular mechanisms that direct the cancer cells toward death (Pileczki et al., 2012). Our results support the ambivalent effects of TNF-α on cancer apoptosis (Kokolakis et al., 2021). Besides, circulating inflammatory cytokines, e.g., TNF-α, IL-6, and IL-10 are discussed as potential prognostic oncomarkers in BC patients due to their contribution to cancer proliferation and invasion (Wang and Yang, 2017; Ahmad et al., 2018). The anticancer activities of *S. officinalis in vivo* and *in vitro* associated with cytokine downregulation in this study, point to a rationale for cytokine blockade in cancer research and clinical practice. In this regard, further evaluation of anti-inflammatory drugs, including phyto-substances, in cancer chemoprevention and treatment is needed.

Alterations in cardinal cellular biomolecules, i.e., DNA, proteins, and lipids may be induced by imbalanced redox status in the cell. Such changes are principally associated with the processes of carcinogenesis (Günes et al., 2020). Antioxidant potential of plant-derived substances may play a prominent role in the prevention of cancer *via* the stable preservation of the mentioned cellular biomolecules. Comprehensive data from our laboratory demonstrated that whole plant foods/substances (applied dietary in relatively low doses), such as young barley leaves, dark fruit peels, clove buds, thyme haulm, cinnamon bark, and sumac fruit, significantly suppressed oxidative damage of lipids or proteins in cancer cells *in vivo* (Kubatka et al., 2016a; Kubatka et al., 2016b; Kubatka et al., 2017b; Kubatka et al., 2019; Kubatka et al., 2020a; Kubatka et al., 2020b). According to the ORAC ranking, salvia belongs among plants with the highest antioxidant capacity (Tundis et al., 2020). With this presumption, we have assessed cellular MDA levels (a well-established marker of oxidative damage of lipids) after salvia treatment. Our data confirmed that salvia at a higher dose decreased MDA levels in tumor cells by 30.5% vs. control. Based on our outcome, we believe that the antioxidant mode of action (geno-protection) typical for salvia represents one of several mechanisms of its anticancer effect found in this study.

Recent scientific data precisely described the role of phytochemicals in targeting specific signaling pathways, e.g., Notch, Wnt, or Hedgehog and *via* this mechanism suppress the vitality of cancer stem cells (CSC) (Liskova et al., 2019; Deldar Abad Paskeh et al., 2021). CSCs play an important role in cancer development by impacting several processes such as apoptosis and the development of drug resistance (Yang et al., 2020). Well-established parameters of BC CSCs include overexpression of CD44^+^/CD24^−/low^ phenotype, EpCAM, CD133, ALDH1, and nestin; all these markers correlate with poorer BC prognosis (Shima et al., 2017). Importantly, CD24, CD44, CD133, EpCAM, and ALDH1 have been shown as valid BC CSCs markers in chemocarcinogen-induced cancer in rodents (Rennó et al., 2015). Salvia in this study showed apparent anti-CSCs activity by reducing expressions of EpCam and ALHDH1 proteins in tumor cells *in vivo*. Previously, we have confirmed the anti-CSC effects of oregano, clove buds, thyme, cinnamon, and sumac in the same animal cancer model (Kubatka et al., 2017a; Kubatka et al., 2017b; Kubatka et al., 2019; Kubatka et al., 2020a; Kubatka et al., 2020b). We assume that the anti-CSCs activities of mentioned medicinal plants are affected *via* the regulation of multiple cell signaling pathways and mechanisms induced by specific mixtures of phytochemicals present in medicinal plants. It seems that the natural cocktails of bioactive molecules demonstrate better oncostatic potential (including the anti-CSC activities) in comparison with the single phytochemicals (Kapinova et al., 2017). In addition, scientific research has revealed anti-CSC effects of plant metabolites not only in BC but also in numerous other cancer types (Liskova et al., 2019). These data demand the need for in-depth analyses and the consequent translation to oncology practice (Naujokat and McKee, 2020).

Abnormal epigenetic modifications including histone chemical alterations, miRNA-modulated post-transcriptional changes, and promoter DNA methylation can alleviate crucial tumor suppressor genes for signal transducers, transcription factors, nuclear receptors, apoptosis-inducing and DNA repair gene products, and cell cycle regulators and thus markedly contribute to initiation and development of cancer (Thakur et al., 2014; Varghese et al., 2020). Aberrant posttranslational chemical modifications of histones were described as factors included in the pathogenesis of numerous types of chronic diseases. In this regard, they could be used as valid prognostic and predictive markers in clinical practice, including oncology (Romanowska and Joshi, 2019). Targeting the histone-modulating enzymes by single phytochemicals or their specific mixtures may provide a novel and progressive medicinal approach to the management of chronic diseases. In this study, salvia in both doses significantly decreased H3K4m3 levels and elevated H4K16ac in rat tumor specimens. Using the same BC model, we revealed beneficial alterations in histone chemical modulations in cancer cells after chronic application of clove buds (Kubatka et al., 2017b), thyme (Kubatka et al., 2019), cinnamon (Kubatka et al., 2020b), and sumac (Kubatka et al., 2020a). In the study of other authors, resveratrol induced cytotoxicity in MDA-MB-231 and MCF-7 cells *via* declining the H4R3me2s and H3K27me3 levels and increasing H3K9ac and H3K27ac levels (Chatterjee et al., 2019). These data correlated with an upregulated BRCA1, p53, and p21 expression. Another *in vitro* study revealed reduced levels of histone deacetylase in MCF-7 and MDA-MD-231 lines after the combined application of sulforaphane and withaferin A. Moreover, upregulated histone methylations were associated with cytotoxic effects, apoptosis, and blocking of the cell cycle in BC cells (Royston et al., 2017). Other authors found that epigallocatechin gallate (EGCG) reactivated silenced tumor suppressor genes *via* decreasing histone deacetylase activity and increasing the acetylation in lysine 9 and 14 on H3 and lysine 5, 12, and 16 on H4 and *via* decreased methylation of lysine 9 on H3 (Nandakumar et al., 2011). Even though our study did not reveal the precise mechanisms by which salvia modulates posttranslational histone modifications in used BC model *in vivo*, the comprehensive preclinical and clinical data point to dietary phytochemicals as a progressive tool to control cancer (Uramova et al., 2018; Samec et al., 2019a). A deeper knowledge of the mechanisms linked with the posttranslational histone modifications may disclose new molecular targets for plant-derived substances, therefore, further research is warranted.

MiRNAs play a crucial role in the modulation of gene expression; therefore, miRNAs are substantially involved in numerous physiological and pathophysiological processes including carcinogenesis. MiRNAs act as either potential oncogenes or tumor-suppressor genes; thus, in many cases, their role in the pathogenesis of BC and clinical utility remains disputable (Zhang et al., 2022). Prognostic miRNA data are crucial for answering very complex questions concerning specific BC patient outcomes. Specific miRNAs have been introduced as valid biomarkers in BC management including miR21, miR155, miR210, miR22, miR34a, and miR145. Specifically, miR21 and miR155 function as oncogenic miRNAs, on the other hand, miR22, miR34a, and miR145 are documented as tumor suppressors in breast carcinogenesis. MiR210 is characterized as oncogenic under hypoxic conditions in the cell (Qin et al., 2014). This study demonstrated significant decreases in oncogenic miR21 and tumor-suppressive miR145 and an apparent (but non-significant) decrease in oncogenic miR155 expression when compared to controls. Significant effects of *T*. *vulgaris*, *C*. *zeylanicum*, and *R. coriaria* on the expression of specific miRNAs in the same BC model were found also in our recent studies (Kubatka et al., 2019; Kubatka et al., 2020a; Kubatka et al., 2020b). Based on not completely coherent data within the comprehensive BC research (including data from our laboratory), robust clinical and preclinical validation focused on a deeper understanding of molecular mechanisms is needed to precisely define the role of specific miRNA signatures and particular miRNAs in BC and its specific subtypes in the context of disease diagnosis, prognosis, and preventive strategies (Zografos et al., 2019).

Evaluating the methylation of the patterns of TSG promoter regions using standardized CpG islands in rat tumor cells *in vivo* treated with salvia showed a significant decline in ATM and PTEN promoters. Considerable, but non-significant decrease in six CpG islands of the TIMP3 promoter region after salvia treatment was found in this study. We have documented similar data using the same chemopreventive model of BC. Natural bioactive metabolites present in Syzygium aromaticum, *T. vulgaris*, C. zeylanicum, and *R. coriaria* initiated significant downregulation in the methylation status of clinically validated TSG promoters (clove: RASSF1; thyme: ATM, RASSF1, PTEN, and TIMP3; cinnamon: ATM and TIMP3; sumac: ATM, PTEN, and TIMP3 (Kubatka et al., 2017b; Kubatka et al., 2019; Kubatka et al., 2020a; Kubatka et al., 2020b). As we documented in our laboratory, demethylating plant-derived substances (as a cocktail of bioactive secondary metabolites in whole plants) *by* affecting the activity of DNA methyltransferases can modulate specific gene expression by reversing the abnormal epigenetic changes that are common cancer inducers. Future oncology research must be unambiguously aimed toward the clinical sphere, applying a more personalized approach to appoint the beneficial role of plant-derived substances as significant epigenetic modulators in cancer or high-risk individuals (Jasek et al., 2019).

Cancer, comprising consecutive genetic and epigenetic modifications, leads to multiple aberrant metabolic routes in the cells and tissues. Comprehensive scientific data supports the potential of phytochemicals to meddle with several key metabolic routes, e.g., tumour glycolysis and the modulation of hypoxia (Pralea et al., 2022; Samec et al., 2023). Zhang et al. highlighted the relevance of BCAA metabolic reprogramming in BC. The authors showed that the proliferation of BC cells through mTOR-modulated mitochondrial biogenesis is stimulated by BCAT transaminase 1, which catalyzes the conversion of BCAAs to BCKAs (Zhang and Han, 2017). In our study, after treatment of animals with induced BC by *S. officinalis*, we observed reduced amounts of all three BCKAs in blood plasma which would be in link with the decrease in BCAT activity in conditions of the suppressed BC growth in rats.

Cancer cells rely heavily on glucose and convert it rapidly to pyruvate via glycolysis. Instead of pyruvate entering the TCA cycle, cancer cells turn most of the pyruvate into lactate which is released into the environment, then reuptaken from circulation and converted back to pyruvate to fuel the TCA cycle. It has been shown that the turnover of circulating lactate is the highest of all metabolites and lactate is the main source for the TCA cycle in normal tissue as well as in tumors (Hui et al., 2017). In the SAL 0.1 group, blood plasma levels of lactate and pyruvate were observed to be decreased in comparison to the non-treated group, suggesting less metabolically active tumorous tissue. The lowered levels of pyruvate influence the functioning of TCA which is manifested in decreased citrate plasma levels. The importance of metabolites involved in energy energy-gaining TCA cycle in comprehensive cancer development and progression was demonstrated also for succinate, accumulated in blood plasma in the SAL 0.1 group. Succinate is interconnected with inflammatory responses in macrophages and other immune cells. In addition to its role in TCA metabolism, succinate acts as a regulatory signal enhancing Il-1β expression and inhibits the hydroxylation of HIF-1α (Cordes et al., 2016). Various mechanisms could be proposed as the cause of succinate accumulation, including increased glutamine anaplerosis and oxidation in the TCA cycle. Just this seems to be likely as we observed in parallel a significant decrease in glutamine levels in the SAL 0.1 group. Although reduced circulating glutamine could be linked to the increased glutamine demand by BC cells, this seems to be improbable regarding applied *S. officinalis* treatment showing cancer-suppressing effects. Instead, in line with increased succinate, reduced glutamine levels are rather associated with enhanced immune response, since glutamine is known to serve as an essential fuel for all immunocompetent cells. On the other hand, in the SAL 1 group, the accumulation of circulating pyruvate was observed, suggesting its decreased utilisation, probably due to impaired transport into the mitochondria. The decreased citrate level in this group supports the thesis of the alterations in pyruvate conversion, and together with decreased succinate levels also slowdown of TCA cycle, which is, in contrast to SAL 0.1, not supplemented by circulating glutamine. In both groups, observed changes are likely linked with the phytopharmacological effect of *S. officinalis* affecting the AcetylCoA biosynthesis which is an important molecule that connects lipid metabolism with histone acetylation, forms a more complex regulatory mechanism that influences cancer growth, proliferation, and metastasis (He et al., 2023).

Finally, SAL 0.1 rats showed decreased plasma levels of essential amino acid histidine, suggesting accelerated histamine production. Histamine is a molecule with dual effects in carcinogenesis: although some studies support its pro‐tumorigenic effects, many experimental and clinical findings show that it is a crucial mediator of immune cell responses, participating in anti-tumour immunity (Nguyen and Cho, 2021). Considering the other results from our work, we believe that histidine depletion in SAL 0.1 animals may participate in the antitumorous effect of phytopharmacological treatment, although the exact mechanism is not known. SAL 0.1 animals also showed accelerated utilization of circulating tyrosine, which is a precursor of dopamine and other catecholamines, which were reported to exhibit an inhibitory effect on tumour growth in the breast (Jayachandran et al., 2023). In summary, observed plasma metabolic changes in BC rats treated group with *S. officinalis* did not follow the same pattern in both doses, where differences, particularly in levels of metabolites involved in energy metabolism, may be linked with additional, but so far unknown effects of phytochemicals on plasma metabolome.

## 5 Conclusion

Our study assessing the anti-cancer effects of *S. officinalis* haulm, using BC animal models and *in vitro* approach, is the first to provide comprehensive preclinical research data. *S. officinalis* in both rodent BC models (allograft mouse and chemically-induced carcinogenesis in rats) showed significant anti-cancer activities. The beneficial effects of salvia were associated with significant changes in the histopathology of tumors observed in rodent models. Assessing the mechanism of action, using *in vivo* and *in vitro* experimental approaches, revealed proapoptotic and antiproliferative effects. Besides, salvia haulm showed increased antioxidant activity and decreased stemness, and positive epigenetic and metabolic alterations in BC cells *in vivo*. These positive changes in apoptosis, CSC markers, and epigenetics were implemented in a significant prolongation of tumor latency and a significantly improved prognosis of tumors in rats treated with salvia. Our data revealed the initiation of non-specific cellular signaling that was associated with apparent anticancer activities of salvia against BC models in our study. In this regard, systemic in-depth analyses and an understanding of this signaling network are needed within translational research. Importantly, single plant-derived molecules or their natural mixtures present in whole foods demonstrate significant anti-cancer effects, however, they have not yet been applied in the clinical management of BC. For this reason, their application within novel treatment strategies and/or chemoprevention of BC needs continuing clinical evaluations and resolving several important issues such as (a) defining pharmacokinetics linked with effective and safe dosing and mode of administration, (b) specification of sensitive cancer types concerning individual characteristics of patients, and (c) determination of suitable combined clinical applications with conventional drugs to re-sensitize cancer cells.

## Data Availability

The original contributions presented in the study are included in the article/supplementary materials, further inquiries can be directed to the corresponding authors.
